# Quantitative HDL Proteomics Identifies Peroxiredoxin-6 as a Biomarker of Human Abdominal Aortic Aneurysm

**DOI:** 10.1038/srep38477

**Published:** 2016-12-09

**Authors:** Elena Burillo, Inmaculada Jorge, Diego Martínez-López, Emilio Camafeita, Luis Miguel Blanco-Colio, Marco Trevisan-Herraz, Iakes Ezkurdia, Jesús Egido, Jean-Baptiste Michel, Olivier Meilhac, Jesús Vázquez, Jose Luis Martin-Ventura

**Affiliations:** 1 Vascular Research Lab, IIS-Fundación Jiménez Díaz-Autonoma University, Madrid, Spain; 2 Cardiovascular Proteomics Laboratory, Centro Nacional de Investigaciones Cardiovasculares (CNIC), Madrid, Spain; 3 Centro de Investigación Biomédica en Red de Diabetes y Enfermedades Metabólicas Asociadas (CIBERDEM), Spain; 4 Inserm, U698, Universite Paris 7, CHU X-Bichat, Paris, France; 5 Diabète athérothrombose Thérapies Réunion Océan Indien (UMR DéTROI U1188) – Université de La Réunion-CYROI- 2, rue Maxime Rivière 97490 Sainte Clotilde – La Réunion – France

## Abstract

High-density lipoproteins (HDLs) are complex protein and lipid assemblies whose composition is known to change in diverse pathological situations. Analysis of the HDL proteome can thus provide insight into the main mechanisms underlying abdominal aortic aneurysm (AAA) and potentially detect novel systemic biomarkers. We performed a multiplexed quantitative proteomics analysis of HDLs isolated from plasma of AAA patients (N = 14) and control study participants (N = 7). Validation was performed by western-blot (HDL), immunohistochemistry (tissue), and ELISA (plasma). HDL from AAA patients showed elevated expression of peroxiredoxin-6 (PRDX6), HLA class I histocompatibility antigen (HLA-I), retinol-binding protein 4, and paraoxonase/arylesterase 1 (PON1), whereas *α*-2 macroglobulin and C4b-binding protein were decreased. The main pathways associated with HDL alterations in AAA were oxidative stress and immune-inflammatory responses. In AAA tissue, PRDX6 colocalized with neutrophils, vascular smooth muscle cells, and lipid oxidation. Moreover, plasma PRDX6 was higher in AAA (N = 47) than in controls (N = 27), reflecting increased systemic oxidative stress. Finally, a positive correlation was recorded between PRDX6 and AAA diameter. The analysis of the HDL proteome demonstrates that redox imbalance is a major mechanism in AAA, identifying the antioxidant PRDX6 as a novel systemic biomarker of AAA.

Abdominal aortic aneurysm (AAA) is a major health problem, with a prevalence of ~2% in adults aged over 65 years^1^,^2^ and causing about 1–2% of male deaths in economically developed societies^3^. Clinically, AAA is defined as a permanent dilation of the aortic diameter by more than 3 cm or more than 50% of the initial value. Mechanistically, AAA is characterized by the formation of an intraluminal thrombus (ILT), proteolysis, oxidative stress, immune inflammatory response, angiogenesis and fibrosis. Currently, the only way to prevent aortic rupture in patients with an AAA >5.5 cm is surgery. AAA is usually asymptomatic and is often detected as an incidental finding during the investigation of an unrelated problem or as a consequence of radiological screening. Moreover, diameter growth is discontinuous, with periods of growth alternating with periods of stability, making prognosis difficult^4^. There is thus a pressing need to identify novel biomarkers of the presence and evolution of AAA, which provide insight into the pathological mechanisms of the disease.

The most convenient source of a systemic AAA biomarker is blood plasma. Our group and others have previously analyzed AAA patient plasma using a variety of proteomics approaches^5^,^6^,^7^,^8^,^9^. However, the high dynamic range of protein concentrations in plasma makes it difficult to quantify proteins present in low amounts, even after depletion of the most abundant proteins. To circumvent this problem, some authors have concentrated on the analysis of specific plasma subproteomes, such as high-density lipoproteins (HDLs).

HDLs are a transport platform of lipids and proteins. HDLs are responsible for transporting excess cholesterol from peripheral tissues to the liver for its elimination in feces and bile. Clinical and epidemiological studies consistently link low HDL cholesterol levels to an elevated risk of cardiovascular disease. A recent meta-analysis also revealed a negative association of HDL cholesterol levels with AAA in most studies^10^. HDLs are additionally a transport platform for constitutive and non-permanently associated plasma proteins. Screening of the HDL proteome has identified dramatic protein alterations in a variety of disease contexts, including cardiovascular diseases^11^,^12^,^13^,^14^,^15^. HDLs have several important cardiovascular protective properties, including anti-oxidant, anti-inflammatory, and antithrombotic effects^16^,^17^. These properties are attributable to HDL-associated proteins, and it is increasingly accepted that HDL composition, rather than quantity, is more important for its vasculo-protective activities. We previously demonstrated impaired anti-proteolytic^18^ and anti-oxidative functions^19^ in HDLs from human AAA. This effect has been linked to altered HDL protein composition or to impaired function of individual components. However, the protein alterations taking place in the HDL proteome in AAA have not been reported to date.

Here, we performed a multiplexed quantitative proteomics study of HDL-protein alterations in AAA. Systems biology analysis revealed an association of AAA with an increased HDL content of proteins related to redox homeostasis, most clearly evident in the increased levels of peroxiredoxin-6 (PRDX6). Circulating levels of PRDX6 are also increased in AAA-patient plasma, and PRDX6 levels correlate positively with AAA size, identifying PRDX6 as a promising biomarker of AAA.

## Results

### Analysis of the human HDL proteome in AAA

**Figure 1:**
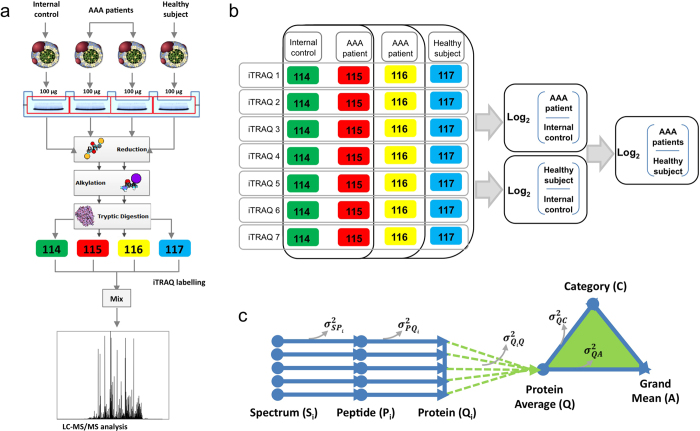
Multiplexed analysis of individual HDL proteomes from AAA patients: shotgun proteomics workflow. (**a**) HDL particles were isolated by sequential ultracentrifugation. Protein extracts were loaded onto SDS-PAGE gels, and proteins were concentrated in the stacking gel. After in-gel trypsin digestion, peptides were labeled with 4-plex iTRAQ reagents and then analyzed by LC-MS/MS. (**b**) A total of 7 iTRAQ experiments were performed. In each experiment, samples from 2 AAA patients and 1 control subject were compared with an internal control. The internal control was a pool of all the individual samples used in the study. (**c**) The statistical model for the quantitative data decomposes the total technical variance into the spectral, peptide, and protein variance components and provides a general framework to fully integrate quantitative and error information, allowing a comparative analysis of the results obtained from the 7 iTRAQ experiments.

HDL particles were isolated from plasma and subjected to trypsin digestion. The resulting peptides were iTRAQ-labeled and combined in 7 independent experiments for LC-MS/MS analysis (Fig. 1). The protein composition of the HDL samples was characterized by spectral counting of the 7 HDL pools. At a 1% FDR threshold, 112 proteins were identified as HDL particle constituents in at least 3 experiments (Supplementary Table S1). As expected, ApoA1 was one of the most abundant HDL proteins, representing 33% of total protein composition. Other abundant HDL proteins were *α*-1-antitrypsin, complement C3, apolipoproteins D, E and M, and paraoxonase/arylesterase 1 (3–4% each) (Supplementary Figure S1). Albumin and ApoB100 were also identified but were not included in the analysis because they are abundant plasma proteins and are considered concomitant contaminants of HDLs obtained by ultracentrifugation^12^. Of the proteins identified in this study, 78 have previously been described as HDL-associated (Supplementary Table S1). These proteins comprise 92% of the absolute HDL protein content. A further 34 HDL proteins identified here, representing 8% of total HDL composition, have not been previously linked to HDL (Supplementary Table S1).

**Figure 2:**
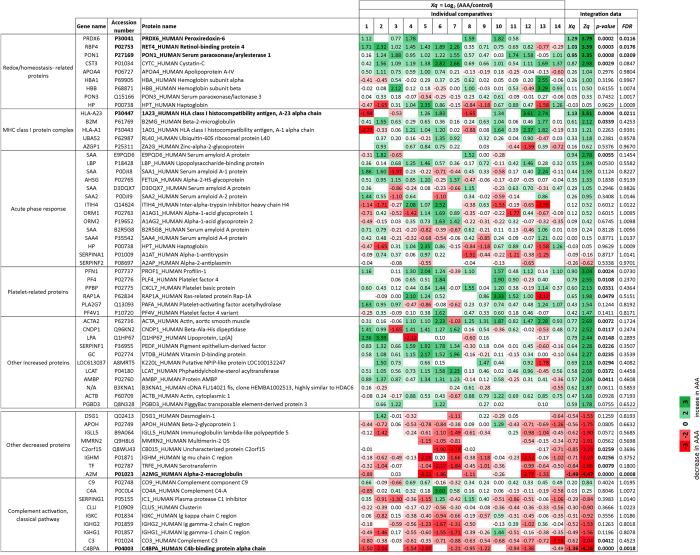
Protein abundance changes in the HDL proteome of AAA patients. Quantitative data for proteins belonging to significantly altered categories according to the colour scale at the bottom. Statistically significant changes (*p-values* < 0.05; *FDR* < 0.05) are shown in bold.

The complete list of quantitative results at the protein level is presented in Supplementary Table S2. Within the set of proteins found in at least 3 iTRAQ experiments, *α*-2 macroglobulin and C4b-binding protein were the two proteins showing the most prominent abundance deficit in HDL from AAA patients compared with controls, whereas PRDX6, HLA-I, retinol-binding protein 4, and PON1 were the proteins showing the largest abundance increases (Fig. 2).

**Figure 3:**
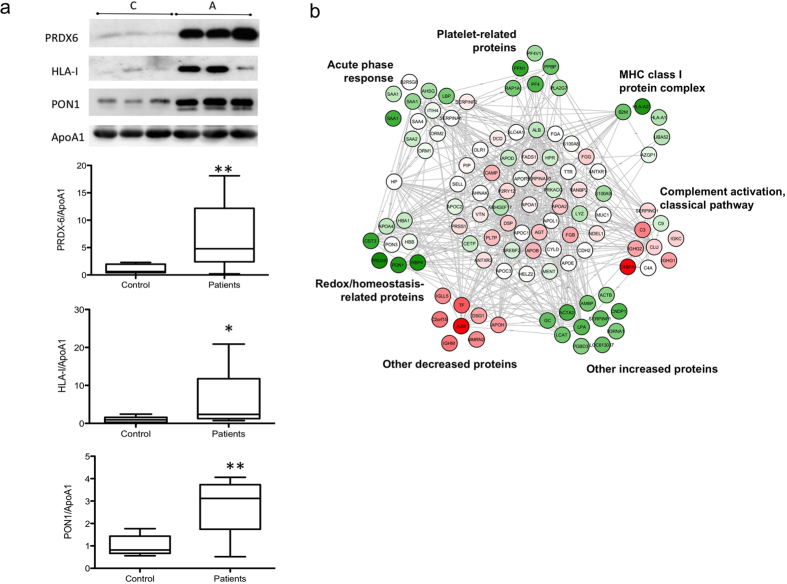
Validation and interaction network of the AAA HDL proteome. (**a**) Representative protein validation by western blot. The quantitative results for PRDX6, HLA-I, and PON1 correspond to the same 14 AAA and 7 control samples used in the proteomic analysis. *p < 0.05, **p < 0.01. (**b**) Interaction network of HDL proteins showing the quantitative data. Significant category changes were clustered.

Among the identified proteins, PRDX6 attracted our interest because it showed the most significant difference between AAA patients and controls and had not previously been linked to HDL or AAA. The quantitative proteomics results for PRDX6 were therefore validated by western blot in the same 14 AAA and 7 control samples (Fig. 3a and Supplementary Figure S2). Western blot was also used to validate HLA-I and PON1, which were also not previously linked to AAA (Fig. 3b and Supplementary Figure S2).

To explore whether the observed protein changes were discrete events or part of a more general pattern of HDL protein alterations, we performed a systems biology analysis using the SBT model, a statistical framework that captures functional alterations produced by coordinated protein abundance changes^20^. SBT analysis was performed on the averaged protein quantifications obtained from the integration of the experiments. We detected significant increases in proteins related to redox homeostasis (*p* = 0.00001, *FDR* = 0.00045), MHC class I complex (*p* = 0.00178, *FDR* = 0.01386), acute phase response (*p* = 0.03597, *FDR* = 0.06570), and platelets (*p* = 0.00002, *FDR* = 0.00076), whereas complement activation proteins from the classical pathway were decreased (*p* = 0.00042, *FDR* = 0.00787) (Fig. 2). With the exception of *α*-2 macroglobulin, all the proteins detected individually as being significantly altered were found among these altered functional categories, suggesting that they were the most evident consequence of a general reconfiguration of the HDL proteome. This interpretation was reinforced by network analysis, which indicated that most of the altered HDL proteins were interconnected in a dense interaction network containing most of the quantified HDL proteins (Fig. 3b).

### Tissue expression of PRDX6 in AAA

**Figure 4:**
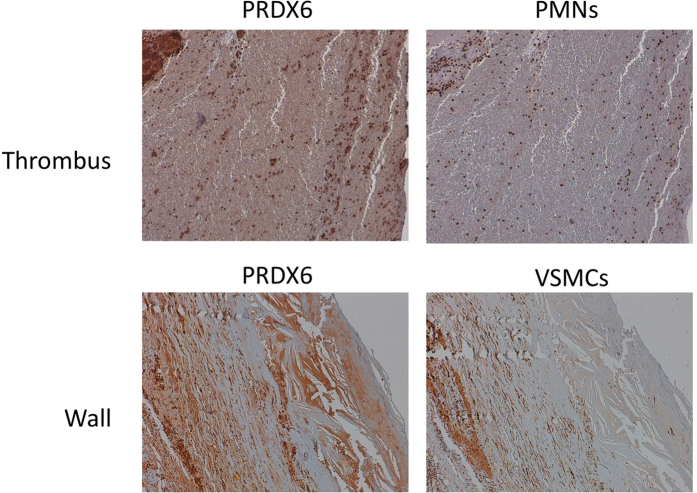
PRDX6 immunohistochemistry in AAA tissue. PRDX6 was detected in AAA thrombus (colocalizing with neutrophils: PMNs, CD15 staining) and wall (colocalizing with vascular smooth muscle cells: VSMC, alpha actin staining). Magnification x10. N = 10.

**Figure 5:**
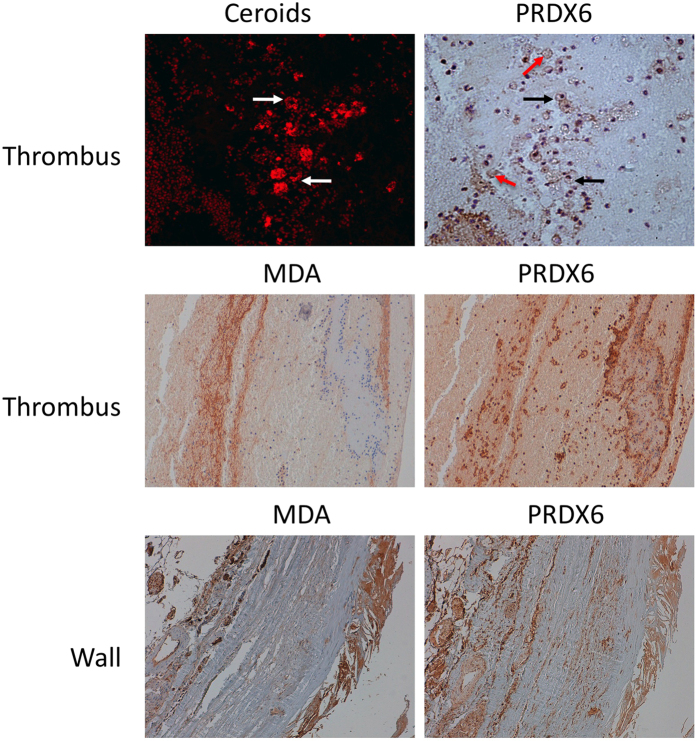
PRDX6 is expressed in areas of high oxidative stress in AAA tissue. PRDX6 colocalizes with ceroids and the lipid peroxidation marker MDA in AAA thrombus and wall. Black arrow indicates typical ceroid rings; red arrow indicates degenerated red blood cells. Magnification x10 (x40 in the upper part of ceroids and PRDX6). N = 10.

Of the validated proteins, we focused on PRDX6 because it reflects the generalized alteration in proteins implicated in redox homeostasis, the functional category most clearly changed in the HDL proteome of AAA patients. Immunohistochemical analysis of PRDX6 expression and localization in the ILT obtained from AAA patients revealed intense PRDX6 immunostaining associated with areas of neutrophil infiltration and RBC degeneration (Figs 4 and 5). Examination of the aortic wall of AAA patients showed strong PRDX6 staining in cholesterol-rich and acellular atherosclerotic plaques in the media layer, but also revealed PRDX6 colocalization with vascular smooth muscle cells (VSMCs) (Fig. 4). PRDX6 also colocalized in AAA tissue with the lipid peroxidation marker MDA and with ceroids, markers of RBC-associated oxidation (Fig. 5).

### Circulating PRDX6 in AAA patients

**Figure 6:**
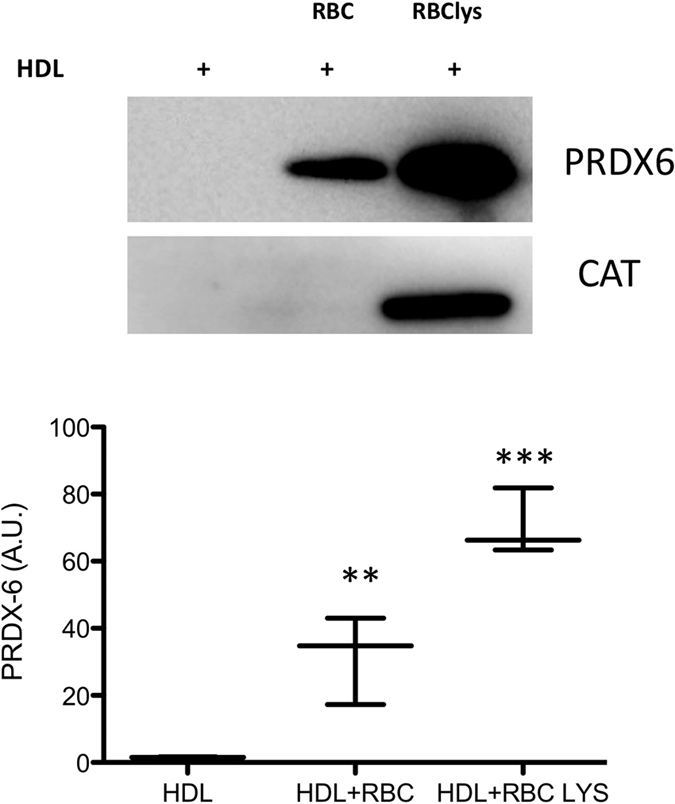
PRDX6 protein levels in HDL after incubation with RBCs. Western blot of PRDX6 and catalase (CAT) in HDL samples incubated with red blood cells (RBC) and lysed RBCs (RBClys). Quantification of densitometric analysis is shown (N = 3). **p < 0.01, ***p < 0.001.

The association of PRDX6 with circulating HDL particles could arise from interaction of HDL particles with circulating cells or from direct loading from plasma. The localization of PRDX6 in areas of erythrophagocytosis suggested that the presence of PRDX6 in HDL particles could arise, at least in part, from the interaction of HDLs with circulating RBCs. To test this hypothesis, we incubated HDLs isolated from healthy subjects with intact or lysed RBCs, and then re-isolated the HDLs to analyse PRDX6 content. PRDX6 levels were low in untreated HDLs from healthy subjects, but increased markedly when incubated with intact or lysed RBCs (Fig. 6). In control experiments, the abundant RBC protein catalase was taken up by HDL particles incubated with lysed RBCs but not by particles incubated with intact RBCs.

**Figure 7:**
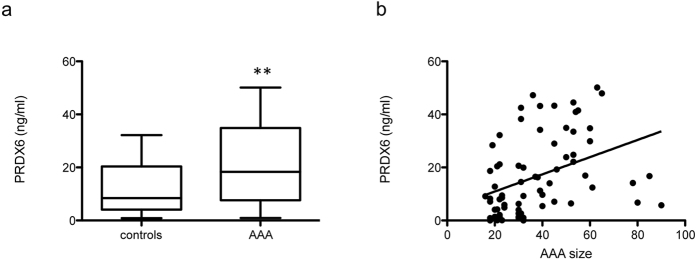
ELISA analysis of PRDX6 in AAA patient plasma. (**a**) Plasma concentration of PRDX6 in a cohort of control participants (N = 27) and AAA patients (N = 47)(**p < 0.01). (**b**) Correlation between PRDX6 and AAA size (r = 0.4, p < 0.001, age-adjusted).

Given that HDL is a platform of plasma-associated proteins, we also examined whether PRDX6 is present in plasma and could serve as a circulating biomarker of AAA. The plasma concentration of PRDX6 was analyzed in a cohort of control individuals (N = 27) and AAA patients (N = 47). Cohort clinical characteristics are shown in Table 1. This analysis confirmed the presence of PRDX6 in plasma, which to our knowledge has not been reported before, and also revealed higher plasma PRDX6 levels in AAA patients than in controls (21 ± 2 vs 10 ± 2 ng/mL; p < 0.01) (Fig. 7a). Multivariate logistic regression analysis of AAA risk factors revealed plasma PRDX6 concentration, hypertension, and smoking to be independent predictors of the presence of AAA [Odds Ratio CI (95%): 1.060 (1.006–1.117), p < 0.05 for PRDX6; 5.775 (1.670–19.973), p < 0.01 for hypertension; and 10.028 (1.139–88.311), p < 0.05 for smoking)]. Furthermore, plasma PRDX6 level correlated positively with AAA size (r = 0.4, p < 0.001, adjusted for age) (Fig. 7b), reinforcing the potential of PRDX6 as a biomarker of AAA.

## Discussion

HDLs are a complex and heterogeneous family of particles with different lipid and protein cargo and functionality. Different methodologies can be used to isolate HDLs, and not all HDL subpopulations are equivalent. In this study, we isolated HDLs by the well-established ultracentrifugation method to isolate most particle subclasses and obtain a general proteomics view of HDLs^21^. However, it is important to note that the small size of HDLs means that all the proteins detected and quantified by mass spectrometry are unlikely to reside in the same particle simultaneously. HDLs are currently regarded as a transport platform of constitutive and non-permanently associated lipids and proteins that may have specific functions in disease^17^. Our analysis identified and quantified 112 proteins that are consistently associated with HDLs in AAA, of which 34 proteins (8% of HDL by composition) have not been previously characterized as HDL components. Quantitative proteomics demonstrated that HDLs in AAA patients are particularly enriched in PRDX6, HLA-I, retinol-binding protein 4, and PON1 and are depleted in C4b-binding protein alpha chain and *α*-2 macroglobulin, among others. Systems biology analysis demonstrated that these prominent changes reflect a general increase in proteins related to redox homeostasis, acute-phase response, and platelet activation and a decrease in proteins related to complement activation.

The levels and functionality of HDLs are altered in immune inflammatory diseases such as rheumatoid arthritis and systemic lupus erythematosus (SLE)^22^. Interestingly, serum amyloid A, one of the HDL components we found to be increased in AAA, has been suggested to underlie the impaired anti-inflammatory properties of HDL in SLE patients^23^, and lack of endogenous acute-phase serum amyloid A protects against experimental AAA^24^. We also found that AAA patients have below-normal levels of C3, consistent with our previous finding that C3 declines in AAA patient plasma, contrasting its accumulation, consumption, and activation in the AAA thrombus^25^. In addition to the known role of HDLs in the humoral response through modulation of the complement system, HDL particles may also influence the innate and adaptive immune responses by modulating antigen presentation functions in macrophages, B cells, and T cells^26^. In line with this idea, we found that HDLs from AAA patients have increased levels of HLA-I, a central immune system molecule that presents endoplasmic-reticulum-derived peptides. The identification of HLA-I in HDL supports the role of HDL as a platform for the assembly of innate immune complexes^27^. Wang *et al*. demonstrated that dysfunctional HDLs promote lipid raft disruption, resulting in less control of antigen presentation and T cell activation^28^. Together, these results support an important role for HDLs in innate and adaptive immune responses, not only as a passive transport platform, but also through the active contribution of constituent proteins.

Proteins with roles in redox balance have been previously observed in HDLs^12^. Here, PON1 and PRDX6, two proteins implicated in oxidative stress, were increased in HDL from AAA patients. PON1 is known for its HDL-associated antioxidant capacity^29^. Vaisar *et al*.^12^ reported elevated HDL PON1 content in patients with coronary artery disease, fitting well with our observation in AAA patients. However, we recently found that serum PON1 activity is decreased in AAA patients^30^, suggesting that PON1 activity is impaired in HDLs of AAA patients. PRDX6 belongs to the peroxiredoxin family, a set of enzymes implicated in the protection against oxidation and the control of H_2_O_2_ signaling^31^. Other members of the PRDX family have been previously associated with AAA, such as PRDX-1 and PRDX-2^32^,^33^. However, this is the first time that PRDX6 has been associated with both HDL and AAA. PRDX6 is a bifunctional enzyme, with glutathione peroxidase and phospholipase A2 (PLA2) activities. Because of this dual function, the precise role of PRDX6 is not yet completely understood. Similar to other PRDXs, PRDX6 is able to reduce short-chain hydroperoxides through its peroxidase activity. However, the PLA2 activity specific to PRDX6 has been linked not only to antioxidant properties^34^ but also to pro-oxidant properties^35^. Very recently, PRDX6 expression was shown to support higher Nox1-derived superoxide production, which was reduced by an inhibitor of PRDX6 phospholipase A2 activity^36^. The impaired antioxidant function reported in HDLs from AAA patients^19^ will likely be the result of the imbalance between the levels and activities of the various pro- and antioxidant proteins, including PRDX6.

Previous studies of AAA tissue have shown increased levels of various proteins involved in redox balance. Elevated levels have been reported of the antioxidant proteins thioredoxin-1, PRDX1, and catalase in AAA thrombus, associated with both RBCs and neutrophils^37^. In the present study, high PRDX6 levels were also observed in AAA thrombus, mainly in areas of degenerated RBCs (in the process of erythrophagocytosis) and neutrophils. It is important to note that PRDX6 relocates to the cell membrane during neutrophil activation^38^, which is required for optimal NADPH oxidase activity. The localization of PRDX6 in VSMCs of the AAA wall is probably a response to increased oxidative stress^39^, since PRDX6 expression in VSMCs is known to increase in response to H_2_0_2_^40^. Other oxidative stress markers identified in AAA tissue include the lipid peroxidation marker MDA and ceroids, which mark oxidation associated with lipids and RBCs^19^. We detected colocalization of PRDX6 with ceroids and MDA in the AAA thrombus; moreover, an intense PRDX6 signal was detected in cholesterol-rich and acellular atherosclerotic plaques in the AAA wall, colocalizing with MDA-positive areas. Cell-associated PRDX6 thus might participate in redox imbalance in AAA tissue through protective antioxidant functions or deleterious neutrophil-dependent NADPH activation; however, in a highly oxidative environment such as AAA, PRDX6 may lose its antioxidant activities through oxidative modification by lipid hydroperoxides^41^. Whether PRDX6 plays a protective or deleterious role in AAA tissue deserves further investigation.

Our finding that *ex-vivo* incubation of HDLs with lysed RBCs increases HDL levels of PRDX6 and catalase suggests that part of the PRDX6 observed in AAA thrombus may arise from RBC lysis. However, increased levels of PRDX6, but not catalase, were also observed in HDLs incubated with intact RBCs, suggesting that HDLs may interact directly with membrane-bound PRDX6 in RBCs. In any case, PRDX6 translocation to the cell membrane is important for reactive oxygen species production through NADPH oxidase 2 complex activation^34^,^38^, suggesting that the elevated PRDX6 levels in circulating HDL of AAA patients reflect an increased systemic oxidative stress. Our study not only identifies PRDX6 as a HDL constituent, but also shows that PRDX6 concentration doubles in the plasma of AAA patients, probably reflecting the systemic response to increased oxidative stress in AAA. We also found a positive correlation between PRDX6 and AAA size, a marker of AAA progression and the clinical parameter used in the management of AAA patients.

Our analysis suggests that the altered protein profile of HDLs in AAA reflects disease events, including an increase in antioxidant proteins probably associated with a systemic response to the redox imbalance in AAA. The increased HDL and plasma levels of PRDX6 in AAA patients support the potential of PRDX6 as a new biomarker of AAA.

## Methods

The authors declare that all methods were performed in accordance with the relevant guidelines and regulations.

### Patient selection

The studies were approved by the Research and Ethics Committee of the Fundación Jiménez Díaz University Hospital Health Research Institute (IIS-FJD; Madrid, Spain), and patients and control participants gave informed consent for their inclusion in the study. Patients with an asymptomatic infrarenal AAA (aortic size >3 cm confirmed by abdominal ultrasound) were recruited during clinical examination or before surgical repair at the Vascular Surgery Service at FJD University Hospital. Controls with non-dilated infrarenal aortas (aortic size <3 cm, confirmed by abdominal ultrasound) were recruited through a screening program. For proteomic analysis, EDTA plasma samples were obtained from 14 male AAA patients and 7 male control participants. For ELISA, a second set of plasma samples (27 controls and 47 AAA patients) were obtained from the IIS-FJD biobank. Clinical characteristics of all controls and AAA patients are summarized in Table 1. For immunohistochemistry, samples of AAA thrombus tissue (n = 10) and wall tissue (n = 10) were collected from male patients (70 ± 6 years old, 70% hypertensive, 30% current smokers, 50% dyslipidemic, 10% diabetic, 20% heart disease) undergoing open surgical repair due to aortic dilation >5 cm at the IIS-FJD Vascular Surgery Service.

### HDL isolation

Lipoproteins were isolated from individual EDTA plasma samples by ultracentrifugation as described in Supplementary Information. Moreover, additional HDLs were isolated from healthy volunteers for incubation with red blood cells (RBC)(N = 3). Briefly, HDLs were incubated with RBCs (intact or lysed with H_2_O/NaCl) for 4 hours at 37 °C, and HDLs were subsequently re-isolated by ultracentrifugation.

### Proteomics

Proteomic analysis was performed on HDL particles isolated from 14 AAA patients and 7 controls (Table 1). HDL samples were in-gel digested overnight at 37 °C with sequencing-grade trypsin (Promega, Madison, WI, USA) at an 8:1 protein:trypsin (w/w) ratio in 50 mM ammonium bicarbonate, pH 8.8^42^. The resulting peptides were desalted on C18 Oasis cartridges (Waters Corporation, Milford, MA, USA) using 50% acetonitrile (ACN) (v/v) in 0.1% trifluoroacetic acid (v/v) as eluent, and vacuum dried. A total of 7 independent isobaric tags were performed for relative and absolute quantitation (iTRAQ) 4-plex experiments. iTRAQ labeling was performed essentially according to the manufacturer’s instructions, as previously described in detail^42^,^43^,^44^ (Supplementary Information). In each experiment, samples from 2 AAA patients and 1 control participant were compared with an internal control, prepared by pooling protein extracts from all subjects of the study. All the comparisons included independent biological preparations, making a total of 14 comparisons between AAA samples and the internal control sample and 7 comparisons between control samples and the internal control sample. The use of the internal control allowed comparison of data from different individuals in different experiments.

The tryptic peptide mixtures were subjected to nano-HPLC (Easy nLC 1000 liquid chromatograph, Thermo Scientific, San Jose, CA, USA) coupled to a Q Exactive mass spectrometer (Thermo Scientific). Peptides were suspended in 0.1% formic acid, loaded onto a C18 RP nano-precolumn (75 μm I.D. and 2 cm, Acclaim PepMap100, Thermo Scientific), and separated on an analytical C18 nano-column (75 μm I.D. and 50 cm, Acclaim PepMap100) in a continuous gradient increasing from 8% to 30% B over 120 min, followed by a rapid increase from 30% to 90% B over 2 min at a flow rate of 200 nL/min. The Q Exactive mass spectrometer was operated in data-dependent mode with a normal FT-resolution spectrum (70,000 resolution) in the mass range of *m/z* 390–1500, followed by acquisition of data-dependent MS/MS spectra from the 10 most intense parent ions identified in the chromatographic run.

Peptides were identified by searching against a Human Uniprot database supplemented with porcine trypsin (120501 entries; release October 2012). The search was conducted with the SEQUEST algorithm (Proteome Discoverer 1.4, Thermo Finnigan), allowing two missed cleavages and using 600 ppm precursor mass tolerance and 0.03 ppm fragment mass tolerance. Methionine oxidation and cysteine carbamidomethylation were allowed as variable modifications. For peptide iTRAQ labeling, lysine and N-terminal modifications of +144.1020 Da were selected as fixed modifications. The same MS/MS spectra collections were searched against inverted databases constructed from the same target databases. SEQUEST results were analyzed by the probability ratio method^45^; false discovery rates (FDR) for peptide identification were calculated using the refined method^46^. The statistical model used to analyze the quantitative data has been described before in detail^43^ (Supplementary Information). The systems biology analysis was performed using the Systems Biology Triangle (SBT)^20^.

The data set from the analysis of HDL proteome (raw and msf files, protein database fasta file, searching parameters xml file, and excel tables with identification and quantification data) is available in the PeptideAtlas repository (http://www.peptideatlas.org/PASS/PASS00861), which can be downloaded via ftp.peptideatlas.org.

### Western blot

Equal amounts of HDL (20 μg) were loaded onto 10% polyacrylamide gels, electrophoresed and transferred to nitrocellulose membranes. Blots were blocked with 7% died skimmed milk in 0.05% Tris-buffered saline and Tween (TBS-T) for 1 hour and incubated overnight at 4 °C with the following antibodies: anti-PRDX6 (ab16947, abcam), anti-HLA-I (LS-B6775, LifeSpan Biosciences, Inc), anti-paraoxonase (PON1) (ab24261, abcam), anti-ApoA1 (home-made), or anti-catalase (ab52477, abcam). ApoA1 was detected as a loading control. Membranes were washed with TBS-T and incubated with the appropriate secondary antibody (1:2500) for 1 hour at room temperature. After 4 washes, the signal was detected with an ECL chemiluminiscence kit (GE Healthcare).

### Histology and immunohistochemistry

Samples of arterial wall and intra-luminal thrombus obtained from AAA patients were embedded in paraffin, and 4 μm cross-sections were cut. Ceroids were detected by direct observation of tissue by fluorescence microscopy (ceroids autofluoresce at 550 nm, producing a red signal). Immunohistochemistry was performed with antibodies against the following proteins: PRDX6 (ab16947, abcam), the lipid peroxidation marker MDA (ab6463, abcam), the neutrophil marker CD15 (Dako), and alpha smooth muscle actin (Dako). Sections were then incubated with the appropriate biotinylated secondary antibody and ABComplex, followed by staining with 3,30-diaminobenzidine (DAB), hematoxylin counterstaining, and mounting in DPX medium.

### ELISA

The plasma concentration of soluble PRDX6 in AAA and control samples was measured with a commercial ELISA kit (LF-EK0206, AbFrontier).

### Statistical analysis

Data are expressed as mean ± SEM. Between-group comparisons were assessed for categorical variables with the X^2^ test and for numerical variables by Mann-Whitney non-parametric test (ELISA and western blot of controls vs patients) or ANOVA followed by Bonferroni test (western blot of *in vitro* experiment). Multivariate logistic regression analysis included only variables that were statistically significant in the univariate analysis, and was performed to assess predictors of the presence of AAA. Univariate association of PRDX6 with AAA size was assessed by the Pearson correlation test and then adjusted for age. Ninety-five percent confidence intervals (CI) were calculated for each comparison. Differences were considered statistically significant at p < 0.05. Statistical analysis was performed with SPSS 15.0.

## Additional Information

**How to cite this article**: Burillo, E. *et al*. Quantitative HDL Proteomics Identifies Peroxiredoxin-6 as a Biomarker of Human Abdominal Aortic Aneurysm. *Sci. Rep.* **6**, 38477; doi: 10.1038/srep38477 (2016).

**Publisher's note:** Springer Nature remains neutral with regard to jurisdictional claims in published maps and institutional affiliations.

## Supplementary Material

Supplemental Data

## Figures and Tables

**Table 1:** Clinical characteristics of patients and control participants.

iTRAQ HDL	Control (N = 7)	AAA (N = 14)	p-Value
Sex (male/female)	7/0	14/0	ns
Age (years ±SD)	64.9 ± 0.2	66.5 ± 1.2	0.024
Dyslipidemia (%)	64.9	64.9	ns
Current smoker (%)	71.4	50	ns
Diabetes (%)	14.3	14.3	ns
Hypertension (%)	42.9	64.3	ns
Heart Disease (%)	28.6	35.7	ns
Statins (%)	42.9	85.7	0.006
**ELISA PRDX6**	**Control (N = 27)**	**AAA (N = 47)**	**p-Value**
Sex (male/female)	27/0	47/0	ns
Age (years ±SD)	64.9 ± 0.2	66.1 ± 5.5	ns
Dyslipidaemia (%)	55.6	56.5	ns
Current smoker (%)	7.4	38.3	0.03
Diabetes (%)	7.4	19.1	ns
Hypertension (%)	25.9	63.8	0.01
Heart Disease (%)	18.5	17	ns
Statins (%)	11.1	38.3	ns
