# Supplementary material for: Quantitative HDL Proteomics Identifies Peroxiredoxin-6 as a Biomarker of Human Abdominal Aortic Aneurysm
Source: Sci Rep. 2016 Dec 9;6:38477. doi: 10.1038/srep38477 (PMC5146935; doi:10.1038/srep38477)
Supplement: Supplemental Data [file srep38477-s1.pdf]

SUPPLEMENTARY INFORMATION

QUANTITATIVE HDL PROTEOMICS IDENTIFIES PEROXIREDOXIN-6 AS A BIOMARKER OF HUMAN ABDOMINAL AORTIC ANEURYSM

Elena Burillo[1#], Inmaculada Jorge[2#], Diego Martínez-López[1], Emilio Camafeita[2], Luis Miguel Blanco-Colio[1], Marco Trevisan-Herraz[2], Iakes Ezkurdia[2], Jesús Egido[3], Jean-Baptiste Michel[4], Olivier Meilhac[5], Jesús Vázquez[2#] and Jose Luis Martin-Ventura[1#]**

[#] In alphabetical order; these authors contributed equally to this work

Supplementary Methods

HDL isolation

Lipoproteins were isolated from individual EDTA plasma samples by ultracentrifugation. Plasma density was adjusted to ρ=1.063 g/mL with KBr and the sample was overlaid with KBr saline solution (ρ=1.063 g/mL). Samples were ultracentrifuged at 53000 rpm for 18 h at 4°C in a TLA-100 rotor (Beckman). After centrifugation, the upper, apolipoprotein-B-containing fraction was recovered and stored. The bottom fraction, containing HDLs, was adjusted to 1.25 g/mL with KBr and overlaid with KBr saline solution (ρ=1.22 g/mL). The HDL-containing fraction was then ultracentrifuged at 63000 rpm for 24 h at 4°C. After this step, HDLs, in the top layer, were recovered as a single band and were extensively rinsed with saline and concentrated using a centrifugal concentrating device (cutoff 10 kDa). All HDL samples were desalted by centrifugation and 3 washes with saline.

Proteomic study

Experimental design

In each experiment, samples from 2 AAA patients and 1 control subject were compared with an internal control, prepared by pooling protein extracts from all subjects of the study. All the comparatives included independent biological preparations, making a total 14 comparatives between AAA samples vs internal control sample and 7 comparatives between control samples vs internal control sample. The use of internal control allowed comparing data from different individuals in different experiments, as described in the Methods section (FIGURE 1). Only 31 outlier proteins showed significant abundance differences

at a 1% FDR threshold in all comparisons of the 7 integration experiments. Proteins identified in at least three independent comparatives at 1% false discovery rate (FDR) were considered for the analysis. Quantitative data were analysed using the weighted spectrum, peptide, and protein (WSPP) model [1], which allowed decomposition of the total variance into the spectral, peptide, and protein variance components. The distribution of quantitative data at the 3 levels, spectrum, peptide and protein, were in good agreement with the predictions of the statistical model, as depicted from the analysis of sigmoidal distributions obtained in all iTRAQ comparisons (Supplementary Figure S3). The variances at the spectrum and peptide levels were similar in all the pairwise comparisons (Supplementary Table S3), and were within the values expected according to results previously obtained in our laboratory [1-5]. A FDR threshold of 1% was set to detect significant protein-abundance changes from the *Z* values. The high inter-individual differences in protein variance observed in human HDL preparations [4] prompted us to use a shared all-experiment internal control as a reference for quantifications; using this approach, protein variance was consistently below 0.4, a value similar to that we obtained working with pooled human HDL samples [4] or human HDL samples from AAA patients [6] (Supplementary Figure S4).

iTRAQ labelling of peptides

The dried peptides were taken up in 15 µL of iTRAQ solution buffer provided with the iTRAQ kit (ABSciex) and labeled by adding 35 µL of the corresponding iTRAQ reagent in ethanol, followed by 1h incubation at room temperature in 70% ethanol in 180 mM triethylammonium bicarbonate (TEAB), pH 8.53. HDL samples from control subjects were labeled with iTRAQ tags 114, and HDL

samples from AAA patients were labeled with tags 115 and 116. In addition, a pool of all 21 samples was used as an internal control in every iTRAQ experiment and was labeled with tag 117. After quenching with 50 μL 0.5% TFA (v/v) for 30 min, samples were fully dried to completely stop the labeling reaction. The four labeled samples were resuspended in 100 μL 0.1% TFA (v/v), combined in one tube, and cleaned up with C18 Oasis cartridges using 50% ACN (v/v) in 0.1% TFA (v/v) as elution solvent. Samples were dried down prior to LC-MS/MS analysis.

Statistics

The $\log_2$ ratio of concentrations in the two samples being compared, A and B, determined by spectrum *s* of peptide *p* derived from protein *q*, is expressed as $X_{eqps}=\log_2(A/B)$. The $\log_2$-ratio value associated with each peptide, X_{eqp}, is then calculated as a weighted average of the spectra used to quantify the peptide, and the value associated with each protein, X_{eq}, is similarly the weighted average of its peptides. In addition, a grand mean, X_e, is calculated in each experiment as a weighted average of the protein values. The global distribution of values at each level is described using a standardized variable, *Z*, that expresses the quantitative values in units of standard deviation and that in the null hypothesis is expected to follow a N (0,1) normal distribution. Our statistical model was used to average protein quantifications from each comparison between control samples and the internal control and between AAA patient samples and the internal control [1,5]. These averaged protein quantifications yielded integrated data for comparison of AAA patient samples with samples from control participants (FIGURE 1, green arrows), thereby increasing the statistical power to detect protein alterations [1]. Again, the distribution of the

standardized variable describing the variability between different experiments within the same protein was very close to the null hypothesis (Supplementary Figure S3), demonstrating that the quantitative results from all experiments were generally reproducible and therefore can be integrated. The final distribution of averaged protein abundance ratios between AAA patients and controls was also close to that of the null hypothesis, allowing the detection of significant protein abundance changes (Supplementary Figure S3).

Systems biology analysis

Systems biology analysis were performed using the SBT model [5]. Proteins were classified as belonging to a functional *class* or *category*, and statistical analyses were performed to determine the relative abundance of each category by integrating the quantitative values of its protein components, after eliminating protein-category outliers. Proteins were functionally annotated using the Ingenuity Knowledge Database [7,8], CORUM [9], and DAVID [10,11]. Network analysis were obtained using STRINGS [12].

References

1 Navarro, P. *et al.* General statistical framework for quantitative proteomics by stable isotope labeling. *J Proteome Res* **13**, 1234-1247, doi:10.1021/pr4006958 (2014).

2 Bonzon-Kulichenko, E. *et al.* A robust method for quantitative high-throughput analysis of proteomes by 18O labeling. *Mol Cell Proteomics* **10**, M110 003335, doi:M110.003335 [pii] 10.1074/mcp.M110.003335 (2011).

3 Jorge, I. *et al.* Statistical model to analyze quantitative proteomics data obtained by 18O/16O labeling and linear ion trap mass spectrometry: application to the study of vascular endothelial growth factor-induced angiogenesis in endothelial cells. *Mol Cell Proteomics* **8**, 1130-1149, doi:M800260-MCP200 [pii] 10.1074/mcp.M800260-MCP200 (2009).

4 Jorge, I. *et al.* The human HDL proteome displays high inter-individual variability and is altered dynamically in response to angioplasty-induced

atheroma plaque rupture. *J Proteomics* **106,** 61-73, doi:10.1016/j.jprot.2014.04.010 (2014).

5 García-Marqués, F. *et al.* A novel systems-biology algorithm for the analysis of coordinated protein responses using quantitative proteomics. *Mol Cell Proteomics,* doi:10.1074/mcp.M115.055905 (2016).

6 Burillo, E. *et al.* ApoA-I/HDL-C levels are inversely associated with abdominal aortic aneurysm progression. *Thromb Haemost* **113,** doi:10.1160/TH14-10-0874 (2015).

7 Ficenec, D. *et al.* Computational knowledge integration in biopharmaceutical research. *Brief Bioinform* **4,** 260-278 (2003).

8 Calvano, S. E. *et al.* A network-based analysis of systemic inflammation in humans. *Nature* **437,** 1032-1037, doi:10.1038/nature03985 (2005).

9 Ruepp, A. *et al.* CORUM: the comprehensive resource of mammalian protein complexes. *Nucleic Acids Res* **36,** D646-650, doi:10.1093/nar/gkm936 (2008).

10 Huang, d. W., Sherman, B. T. & Lempicki, R. A. Systematic and integrative analysis of large gene lists using DAVID bioinformatics resources. *Nat Protoc* **4,** 44-57, doi:10.1038/nprot.2008.211 (2009).

11 Huang, d. W., Sherman, B. T. & Lempicki, R. A. Bioinformatics enrichment tools: paths toward the comprehensive functional analysis of large gene lists. *Nucleic Acids Res* **37,** 1-13, doi:10.1093/nar/gkn923 (2009).

12 Szklarczyk, D. *et al.* STRING v10: protein-protein interaction networks, integrated over the tree of life. *Nucleic Acids Res* **43,** D447-452, doi:10.1093/nar/gku1003 (2015).

Supplementary Table S1.- Proteins identification in at least three iTRAQ experiments. Number of spectra and peptide for each protein identified at 1% FDR and the percent coverage in either are indicated. Accession number from Uniprot database.

Known proteins previously identified in HDL particle		iTRAQ 1			iTRAQ 2			iTRAQ 3			iTRAQ 4			iTRAQ 5			iTRAQ 6			iTRAQ 7		
Accession №	Protein name	n spectra	n peptides	% coverage	n spectra	n peptides	% coverage	n spectra	n peptides	% coverage	n spectra	n peptides	% coverage	n spectra	n peptides	% coverage	n spectra	n peptides	% coverage	n spectra	n peptides	% coverage
P04114	>sp|P04114|APOB_HUMAN Apolipoprotein B-100 OS=Homo sapiens GN=APOB PE=1 SV=2	726	251	56	753	247	54	642	220	50	619	214	50	588	215	50	723	245	56	833	258	58
P02768	>sp|P02768|ALBU_HUMAN Serum albumin OS=Homo sapiens GN=ALB PE=1 SV=2	390	63	80	425	62	79	361	54	75	456	57	78	483	54	75	355	54	74	316	57	78
P02647	>sp|P02647|APOA1_HUMAN Apolipoprotein A-I OS=Homo sapiens GN=APOA1 PE=1 SV=1	588	42	86	532	42	86	350	38	82	394	35	78	510	36	76	447	40	82	486	40	83
P01009	>sp|P01009|A1AT_HUMAN Alpha-1-antitrypsin OS=Homo sapiens GN=SERPINA1 PE=1 SV=3	69	25	52	64	23	48	58	21	50	70	25	53	72	23	53	61	24	51	45	22	53
P01024	>sp|P01024|CO3_HUMAN Complement C3 OS=Homo sapiens GN=C3 PE=1 SV=2	65	40	29	64	44	32	55	40	27	53	34	25	62	41	30	66	46	34	68	46	34
P05090	>sp|P05090|APOD_HUMAN Apolipoprotein D OS=Homo sapiens GN=APOD PE=1 SV=1	78	13	50	61	12	52	53	10	50	58	9	34	45	11	50	50	12	57	68	11	46
P02649	>sp|P02649|APOE_HUMAN Apolipoprotein E OS=Homo sapiens GN=APOE PE=1 SV=1	56	21	64	54	24	58	69	23	68	43	20	62	42	19	57	35	15	52	68	21	68
O95445	>sp|O95445|APOM_HUMAN Apolipoprotein M OS=Homo sapiens GN=APOM PE=1 SV=2	48	15	70	58	17	65	45	11	61	47	11	57	50	11	59	44	12	65	53	11	65
P27169	>sp|P27169|PON1_HUMAN Serum paraoxonase/arylesterase 1 OS=Homo sapiens GN=PON1 PE=1 SV=3	30	10	46	40	11	53	54	12	56	60	12	56	44	12	56	39	11	56	50	13	53
P08519	>sp|P08519|APOA_HUMAN Apolipoprotein(a) OS=Homo sapiens GN=LPA PE=1 SV=1	69	40	35	60	35	35	27	20	25	38	21	26	26	19	25	34	21	33	26	14	30
P00739	>sp|P00739|HPTR_HUMAN Haptoglobin-related protein OS=Homo sapiens GN=HPR PE=1 SV=2	38	13	45	35	13	41	28	13	41	42	13	41	30	13	45	52	15	47	50	17	48
P06727	>sp|P06727|APOA4_HUMAN Apolipoprotein A-IV OS=Homo sapiens GN=APOA4 PE=1 SV=3	29	16	47	38	20	48	29	17	41	28	17	44	43	18	43	31	13	36	42	20	49
P02652	>sp|P02652|APOA2_HUMAN Apolipoprotein A-II OS=Homo sapiens GN=APOA2 PE=1 SV=1	42	6	42	36	7	47	29	6	45	24	6	47	41	6	47	36	6	46	29	6	47
P0C0L4	>sp|P0C0L4|CO4A_HUMAN Complement C4-A OS=Homo sapiens GN=C4A PE=1 SV=1	21	17	13	17	14	11	22	15	13	16	13	12	25	18	15	36	24	21	37	24	17
P0DJI8	>sp|P0DJI8|SAA1_HUMAN Serum amyloid A-1 protein OS=Homo sapiens GN=SAA1 PE=1 SV=1	30	8	55	29	8	55	35	9	66	16	5	55	12	5	55	37	7	55	9	6	43
P35542	>sp|P35542|SAA4_HUMAN Serum amyloid A-4 protein OS=Homo sapiens GN=SAA4 PE=1 SV=2	26	7	42	38	6	48	15	6	33	15	4	38	22	6	38	21	6	38	22	5	38
B2R5G8	>tr|B2R5G8|B2R5G8_HUMAN Serum amyloid A protein OS=Homo sapiens PE=2 SV=1	15	2	42	25	3	65	4	2	33	8	2	38	26	3	56	30	3	56	25	3	56
P02656	>sp|P02656|APOC3_HUMAN Apolipoprotein C-III OS=Homo sapiens GN=APOC3 PE=1 SV=1	27	6	56	20	6	59	13	3	34	15	4	54	17	6	56	16	4	54	19	4	34
O14791	>sp|O14791|APOL1_HUMAN Apolipoprotein L1 OS=Homo sapiens GN=APOL1 PE=1 SV=5	20	12	36	15	11	23	20	9	17	20	9	17	17	9	27	14	7	17	19	11	32
P55058	>sp|P55058|PLTP_HUMAN Phospholipid transfer protein OS=Homo sapiens GN=PLTP PE=1 SV=1	17	11	25	13	9	23	12	8	22	24	13	22	18	10	22	15	9	25	17	12	24
P10909	>sp|P10909|CLUS_HUMAN Clusterin OS=Homo sapiens GN=CLU PE=1 SV=1	22	9	26	14	6	21	9	7	23	13	9	24	15	11	26	18	10	26	21	8	22
Q9UHG3	>sp|Q9UHG3|PCYOX_HUMAN Prenylcysteine oxidase 1 OS=Homo sapiens GN=PCYOX1 PE=1 SV=3	15	11	25	8	7	18	15	11	25	19	13	28	16	10	27	8	6	19	16	14	36
P02766	>sp|P02766|TTHY_HUMAN Transthyretin OS=Homo sapiens GN=TTR PE=1 SV=1	13	8	69	14	8	69	7	6	49	11	7	65	15	7	65	11	7	65	15	7	65
P02654	>sp|P02654|APOC1_HUMAN Apolipoprotein C-I OS=Homo sapiens GN=APOC1 PE=1 SV=1	15	6	29	11	6	37	10	6	29	10	5	27	10	6	29	11	6	29	16	6	29
P02671	>sp|P02671|FIBA_HUMAN Fibrinogen alpha chain OS=Homo sapiens GN=FGA PE=1 SV=2	10	7	14	11	8	14	10	6	9	6	4	6	9	6	10	14	9	13	14	6	8
P02655	>sp|P02655|APOC2_HUMAN Apolipoprotein C-II OS=Homo sapiens GN=APOC2 PE=1 SV=1	10	5	50	15	6	52	9	3	32	9	3	32	15	5	50	8	4	41	5	4	41
P02765	>sp|P02765|FETUA_HUMAN Alpha-2-HS-glycoprotein OS=Homo sapiens GN=AHSG PE=1 SV=1	12	4	16	14	4	16	7	3	16	7	5	18	18	6	19	4	3	9	7	5	14
P02774	>sp|P02774|VTDB_HUMAN Vitamin D-binding protein OS=Homo sapiens GN=GC PE=1 SV=1	4	3	11	8	6	17	8	7	23	12	9	31	20	9	27	11	8	29	6	6	15
P04180	>sp|P04180|LCAT_HUMAN Phosphatidylcholine-sterol acyltransferase OS=Homo sapiens GN=LCAT PE=1 SV=1	8	7	18	10	6	17	9	7	22	13	7	23	10	7	18	9	5	20	9	6	19
Q15166	>sp|Q15166|PON3_HUMAN Serum paraoxonase/lactonase 3 OS=Homo sapiens GN=PON3 PE=1 SV=3	8	6	23	10	7	27	6	4	16	10	6	19	11	7	30	8	6	23	11	7	23
P01834	>sp|P01834|IGKC_HUMAN Ig kappa chain C region OS=Homo sapiens GN=IGKC PE=1 SV=1	5	4	65	4	3	46	15	5	80	9	3	46	12	5	80	7	4	61	6	4	65
P02753	>sp|P02753|RET4_HUMAN Retinol-binding protein 4 OS=Homo sapiens GN=RBP4 PE=1 SV=3	9	5	30	7	5	30	10	6	44	9	6	41	10	6	36	6	5	36	5	4	25
P36955	>sp|P36955|PEDF_HUMAN Pigment epithelium-derived factor OS=Homo sapiens GN=SERPINF1 PE=1 SV=4	6	6	18	6	6	15	7	6	20	9	7	19	12	7	18	5	5	12	9	6	15
P15144	>sp|P15144|AMPN_HUMAN Aminopeptidase N OS=Homo sapiens GN=ANPEP PE=1 SV=4	1	1	1	1	1	1	21	15	19												
P02760	>sp|P02760|AMBP_HUMAN Protein AMBP OS=Homo sapiens GN=AMBP PE=1 SV=1	9	6	16	10	7	20	7	5	16	7	4	16	5	4	13	6	5	16	6	4	13
P01019	>sp|P01019|ANGT_HUMAN Angiotensinogen OS=Homo sapiens GN=AGT PE=1 SV=1	7	5	13	6	5	13	6	4	11	9	6	17	4	4	12	5	4	11	7	3	11
P55056	>sp|P55056|APOC4_HUMAN Apolipoprotein C-IV OS=Homo sapiens GN=APOC4 PE=1 SV=1	4	4	24	4	3	23	2	2	17	5	4	32	9	5	34	7	7	34	9	6	34
P04004	>sp|P04004|VTNC_HUMAN Vitronectin OS=Homo sapiens GN=VTN PE=1 SV=1	9	4	12	4	3	9	4	3	6	5	4	12	6	4	12	5	4	12	6	3	9
P01857	>sp|P01857|IGHG1_HUMAN Ig gamma-1 chain C region OS=Homo sapiens GN=IGHG1 PE=1 SV=1	3	2	9	3	3	15	8	5	22	4	3	15	6	3	22	6	3	22	8	5	25
P01876	>sp|P01876|IGHA1_HUMAN Ig alpha-1 chain C region OS=Homo sapiens GN=IGHA1 PE=1 SV=2	6	4	18	5	4	22	5	4	18	5	3	12	5	4	18	5	4	18	6	4	18

Known proteins previously identified in HDL particle		iTRAQ 1			iTRAQ 2			iTRAQ 3			iTRAQ 4			iTRAQ 5			iTRAQ 6			iTRAQ 7		
Accession №	Protein name	n spectra	n peptides	% coverage	n spectra	n peptides	% coverage	n spectra	n peptides	% coverage	n spectra	n peptides	% coverage	n spectra	n peptides	% coverage	n spectra	n peptides	% coverage	n spectra	n peptides	% coverage
P02748	>sp|P02748|CO9_HUMAN Complement component C9 OS=Homo sapiens GN=C9 PE=1 SV=2	5	4	8	5	5	12	3	3	6	4	4	9	6	6	13	5	5	11	6	5	11
P02749	>sp|P02749|APOH_HUMAN Beta-2-glycoprotein 1 OS=Homo sapiens GN=APOH PE=1 SV=3	5	5	25	4	3	20	2	2	13	4	3	15	8	6	33	8	5	28	2	2	13
P02775	>sp|P02775|CXCL7_HUMAN Platelet basic protein OS=Homo sapiens GN=PPBP PE=1 SV=3	6	2	23	3	2	23	2	2	19	5	3	30	5	2	23	7	2	23	2	2	23
P68871	>sp|P68871|HBB_HUMAN Hemoglobin subunit beta OS=Homo sapiens GN=HBB PE=1 SV=2	3	3	24	3	3	29	5	3	31	8	5	39	3	3	28	6	5	48	1	1	9
P00738	>sp|P00738|HPT_HUMAN Haptoglobin OS=Homo sapiens GN=HP PE=1 SV=1	5	4	29	4	3	20	4	3	22	4	3	22	4	3	26	3	2	28	3	2	28
Q14624	>sp|Q14624|ITIH4_HUMAN Inter-alpha-trypsin inhibitor heavy chain H4 OS=Homo sapiens GN=ITIH4 PE=1 SV=4	4	4	7	1	1	1	1	1	1	5	5	7	7	5	9	5	5	9			2
P62736	>sp|P62736|ACTA_HUMAN Actin, aortic smooth muscle OS=Homo sapiens GN=ACTA2 PE=1 SV=1	4	4	11	6	5	15	5	5	17	4	3	6	3	3	11	1	1	2	2	2	6
P07737	>sp|P07737|PROF1_HUMAN Profilin-1 OS=Homo sapiens GN=PFN1 PE=1 SV=2	1	1	10				8	5	40	5	4	30	3	2	20	1	1	10	1	1	10
P19652	>sp|P19652|A1AG2_HUMAN Alpha-1-acid glycoprotein 2 OS=Homo sapiens GN=ORM2 PE=1 SV=2	3	2	18	4	2	18	4	2	18	4	3	22	2	2	18	4	2	18	1	1	18
P02679	>sp|P02679|FIBG_HUMAN Fibrinogen gamma chain OS=Homo sapiens GN=FGG PE=1 SV=3	3	3	11	2	2	7	6	5	17	1	1	11	2	1	2						
P02675	>sp|P02675|FIBB_HUMAN Fibrinogen beta chain OS=Homo sapiens GN=FGB PE=1 SV=2	4	4	9	4	3	10	3	3	11	1	1	3	1	1	4	4	4	14	2	2	8
P81605	>sp|P81605|DCD_HUMAN Dermcidin OS=Homo sapiens GN=DCD PE=1 SV=2	2	2	23	1	1	13	6	3	25	5	2	25	2	1	25	1	1	13	2	2	25
P58335	>sp|P58335|ANTR2_HUMAN Anthrax toxin receptor 2 OS=Homo sapiens GN=ANTXR2 PE=1 SV=5	1	1	5	2	2	5				2	2	7	4	2	5	2	1	3	4	2	5
P07988	>sp|P07988|PSPB_HUMAN Pulmonary surfactant-associated protein B OS=Homo sapiens GN=SFTPB PE=1 SV=3	2	2	7	4	3	10	2	2	7	3	3	12	3	3	12	2	2	8	1	1	3
P69905	>sp|P69905|HBA_HUMAN Hemoglobin subunit alpha OS=Homo sapiens GN=HBA1 PE=1 SV=2	1	1	11	1	1	11	3	2	17	6	2	19	1	1	11	3	2	17	2	1	11
P02787	>sp|P02787|TRFE_HUMAN Serotransferrin OS=Homo sapiens GN=TF PE=1 SV=3	4	3	7	1	1	2	2	2	4				2	2	4	2	2	4	3	3	5
Q13790	>sp|Q13790|APOF_HUMAN Apolipoprotein F OS=Homo sapiens GN=APOF PE=1 SV=2	2	1	4	2	2	8	1	1	4	4	1	8	4	1	8	2	1	8	1	1	4
P60709	>sp|P60709|ACTB_HUMAN Actin, cytoplasmic 1 OS=Homo sapiens GN=ACTB PE=1 SV=1	3	2	19	1	1	14	5	4	23	2	2	15	1	1	9	1	1	5	2	2	14
P01871	>sp|P01871|IGHM_HUMAN Ig mu chain C region OS=Homo sapiens GN=IGHM PE=1 SV=3	1	1	4	1	1	4	3	3	8							2	2	10	3	3	8
P05155	>sp|P05155|IC1_HUMAN Plasma protease C1 inhibitor OS=Homo sapiens GN=SERPING1 PE=1 SV=2	2	2	5	2	2	5	2	2	4	2	2	4	2	2	5	2	1	3	1	1	4
Q8TDL5	>sp|Q8TDL5|BPIB1_HUMAN BPI fold-containing family B member 1 OS=Homo sapiens GN=BPIFB1 PE=2 SV=1	2	1	3	2	2	5	1	1	3	2	2	5	2	2	6	3	2	6	1	1	2
P04003	>sp|P04003|C4BPA_HUMAN C4b-binding protein alpha chain OS=Homo sapiens GN=C4BPA PE=1 SV=2	2	2	7	2	2	4				2	2	4	2	2	4				1	1	3
P01023	>sp|P01023|A2MG_HUMAN Alpha-2-macroglobulin OS=Homo sapiens GN=A2M PE=1 SV=3	2	1	1				1	1	1				1	1	2	3	3	3			
P07477	>sp|P07477|TRY1_HUMAN Trypsin-1 OS=Homo sapiens GN=PRSS1 PE=1 SV=1	1	1	8	4	1	8	1	1	8							1	1	8			
P01034	>sp|P01034|CYTC_HUMAN Cystatin-C OS=Homo sapiens GN=CST3 PE=1 SV=1	3	2	19	2	1	8	2	1	8	2	1	8	1	1	8	1	1	8	1	1	8
P01859	>sp|P01859|IGHG2_HUMAN Ig gamma-2 chain C region OS=Homo sapiens GN=IGHG2 PE=1 SV=2	1	1	10			4	2	2	16	3	2	13	2	1	17	1	1	17	1	1	13
P10720	>sp|P10720|PF4V_HUMAN Platelet factor 4 variant OS=Homo sapiens GN=PF4V1 PE=1 SV=1	1	1	14	2	1	14	1	1	14	1	1	14	1	1	14	4	1	14			
P15924	>sp|P15924|DESP_HUMAN Desmoplakin OS=Homo sapiens GN=DSP PE=1 SV=3										1	1	1	2	2	1				2	2	1
P61769	>sp|P61769|B2MG_HUMAN Beta-2-microglobulin OS=Homo sapiens GN=B2M PE=1 SV=1	1	1	8	2	2	27	3	2	27				1	1	8	1	1	8	1	1	8
P25311	>sp|P25311|ZA2G_HUMAN Zinc-alpha-2-glycoprotein OS=Homo sapiens GN=AZGP1 PE=1 SV=2				1	1	5				2	1	5	1	1	5	2	2	10	1	1	5
P61626	>sp|P61626|LYSC_HUMAN Lysozyme C OS=Homo sapiens GN=LYZ PE=1 SV=1				2	2	16	1	1	8	1	1	8	2	2	16				1	1	8
P02763	>sp|P02763|A1AG1_HUMAN Alpha-1-acid glycoprotein 1 OS=Homo sapiens GN=ORM1 PE=1 SV=1	1	1	4	2	2	11	1	1	4	2	2	12	1	1	4	1	1	4			4
P11597	>sp|P11597|CETP_HUMAN Cholesteryl ester transfer protein OS=Homo sapiens GN=CETP PE=1 SV=2				1	1	3	2	1	3	2	1	5	1	1	5	1	1	3	1	1	3
Q9H8L6	>sp|Q9H8L6|MMRN2_HUMAN Multimerin-2 OS=Homo sapiens GN=MMRN2 PE=1 SV=2													2	2	3	1	1	1	1	1	1
Q9UK55	>sp|Q9UK55|ZPI_HUMAN Protein Z-dependent protease inhibitor OS=Homo sapiens GN=SERPINA10 PE=1 SV=1										2	2	7	1	1	2	1	1	4			
P02730	>sp|P02730|B3AT_HUMAN Band 3 anion transport protein OS=Homo sapiens GN=SLC4A1 PE=1 SV=3	1	1	1	1	1	1	1	1	5	3	2	3	1	1	1	1	1	1	1	1	1
P18428	>sp|P18428|LBP_HUMAN Lipopolysaccharide-binding protein OS=Homo sapiens GN=LBP PE=1 SV=3	1	1	2	1	1	2	1	1	2	2	2	5	1	1	2	2	2	5	1	1	2
P08697	>sp|P08697|A2AP_HUMAN Alpha-2-antiplasmin OS=Homo sapiens GN=SERPINF2 PE=1 SV=3	1	1	3				1	1	2				1	1	3						
P49913	>sp|P49913|CAMP_HUMAN Cathelicidin antimicrobial peptide OS=Homo sapiens GN=CAMP PE=1 SV=1	1	1	10	1	1	13	1	1	5	1	1	5	1	1	5	1	1	5			
Q9H6X2	>sp|Q9H6X2|ANTR1_HUMAN Anthrax toxin receptor 1 OS=Homo sapiens GN=ANTXR1 PE=1 SV=2	1	1	2				1	1	2	1	1	2									

Novel proteins identified in HDL particle in this study		iTRAQ 1			iTRAQ 2			iTRAQ 3			iTRAQ 4			iTRAQ 5			iTRAQ 6			iTRAQ 7		
Accession №	Protein name	n spectra	n peptides	% coverage	n spectra	n peptides	% coverage	n spectra	n peptides	% coverage	n spectra	n peptides	% coverage	n spectra	n peptides	% coverage	n spectra	n peptides	% coverage	n spectra	n peptides	% coverage
Q96KN2	>sp|Q96KN2|CNDP1_HUMAN Beta-Ala-His dipeptidase OS=Homo sapiens GN=CNDP1 PE=1 SV=4	9	8	21	8	7	20	1	1	2	9	8	22	7	6	18	9	8	21	12	9	22
Q6U2E9	>tr|Q6U2E9|Q6U2E9_HUMAN C4B1 OS=Homo sapiens GN=C4B PE=4 SV=1	8	4	15	7	4	13	8	4	13	9	4	14	7	4	17	6	4	22	5	4	19
C0JYY2	>tr|C0JYY2|C0JYY2_HUMAN Apolipoprotein B (Including Ag(X) antigen) OS=Homo sapiens GN=APOB PE=4 SV=1	4	2	57	3	2	55	6	2	50	4	2	51	4	2	51	4	2	57	7	2	58
D3DQX7	>tr|D3DQX7|D3DQX7_HUMAN Serum amyloid A protein OS=Homo sapiens GN=SAA1 PE=3 SV=1	4	2	55	2	2	55	12	2	66	3	2	55	4	2	55	4	2	55	3	2	43
P0DJI9	>sp|P0DJI9|SAA2_HUMAN Serum amyloid A-2 protein OS=Homo sapiens GN=SAA2 PE=1 SV=1	3	2	36	6	2	36	5	3	50	2	2	36	1	1	36	2	2	36	2	2	25
P12273	>sp|P12273|PIP_HUMAN Prolactin-inducible protein OS=Homo sapiens GN=PIP PE=1 SV=1							1	1	5	7	4	26	1	1	8						
B9A064	>sp|B9A064|IGLL5_HUMAN Immunoglobulin lambda-like polypeptide 5 OS=Homo sapiens GN=IGLL5 PE=2 SV=2	3	2	14	2	2	14				4	1	7	3	1	7	2	1	7	2	1	7
P02776	>sp|P02776|PLF4_HUMAN Platelet factor 4 OS=Homo sapiens GN=PF4 PE=1 SV=2							2	1	26	2	2	27	3	2	27	3	2	27			
P62987	>sp|P62987|RL40_HUMAN Ubiquitin-60S ribosomal protein L40 OS=Homo sapiens GN=UBA52 PE=1 SV=2							3	2	20	3	2	20	1	1	13	1	1	7	4	2	20
Q02413	>sp|Q02413|DSG1_HUMAN Desmoglein-1 OS=Homo sapiens GN=DSG1 PE=1 SV=2				2	2	3	1	1	1	6	6	8	1	1	2	1	1	2	3	2	3
Q13093	>sp|Q13093|PAFA_HUMAN Platelet-activating factor acetylhydrolase OS=Homo sapiens GN=PLA2G7 PE=1 SV=1	3	3	9	2	2	7	2	2	6	4	4	13	2	2	5	1	1	4	1	1	2
P01892	>sp|P01892|1A02_HUMAN HLA class I histocompatibility antigen, A-2 alpha chain OS=Homo sapiens GN=HLA-A PE=1 SV=1							2	1					2	2	10	2	2	10			
E9PQD6	>tr|E9PQD6|E9PQD6_HUMAN Serum amyloid A protein OS=Homo sapiens GN=SAA1 PE=3 SV=1	1	1	54	1	1	54	6	2	66				1	1	55	1	1	55			30
P30443	>sp|P30443|1A01_HUMAN HLA class I histocompatibility antigen, A-1 alpha chain OS=Homo sapiens GN=HLA-A PE=1 SV=1	2	2	10	1	1	5	2	2	10	3	3	15	1	1	5	2	2	10	2	1	5
F6KPG5	>tr|F6KPG5|F6KPG5_HUMAN Albumin (Fragment) OS=Homo sapiens PE=2 SV=1							4	1	78	1	1	82	1	1	78	1	1	77			
Q8WXW3	>sp|Q8WXW3|PIBF1_HUMAN Progesterone-induced-blocking factor 1 OS=Homo sapiens GN=PIBF1 PE=2 SV=2	2	1	1	2	1	1				1	1	1									
A8K479	>tr|A8K479|A8K479_HUMAN cDNA FLJ77094, highly similar to Homo sapiens apolipoprotein B (including Ag(x) antigen) (APOB)	2	1	52	2	1	51							1	1	47						
Q1HP67	>tr|Q1HP67|Q1HP67_HUMAN Lipoprotein, Lp(A) OS=Homo sapiens GN=LPA PE=2 SV=1	2	1	37	1	1	36			25	1	1	29	2	1	26	2	1	31			
P05109	>sp|P05109|S10A8_HUMAN Protein S100-A8 OS=Homo sapiens GN=S100A8 PE=1 SV=1							1	1	12	2	2	24	2	2	26				1	1	12
P30447	>sp|P30447|1A23_HUMAN HLA class I histocompatibility antigen, A-23 alpha chain OS=Homo sapiens GN=HLA-A PE=1 SV=1	1	1	14				1	1	14				2	1	14	2	1	14			
Q9BUN1	>sp|Q9BUN1|MENT_HUMAN Protein MENT OS=Homo sapiens GN=MENT PE=1 SV=1	2	2	4	2	2	4				1	1	4	1	1	4	1	1	4	2	2	4
A8K2W6	>tr|A8K2W6|A8K2W6_HUMAN cDNA FLJ76248, highly similar to Homo sapiens phospholipase A2	2	2	12	1	1	10							2	1	9	1	1	7			2
P14151	>sp|P14151|LYAM1_HUMAN L-selectin OS=Homo sapiens GN=SELL PE=1 SV=2				1	1	5				1	1	2	1	1	5	3	3	10	1	1	3
P23528	>sp|P23528|COF1_HUMAN Cofilin-1 OS=Homo sapiens GN=CFL1 PE=1 SV=3							2	2	19				1	1	7	1	1	7			
P31944	>sp|P31944|CASPE_HUMAN Caspase-14 OS=Homo sapiens GN=CASP14 PE=1 SV=2										2	1	6	1	1	6				1	1	6
P06702	>sp|P06702|S10A9_HUMAN Protein S100-A9 OS=Homo sapiens GN=S100A9 PE=1 SV=1				1	1	13				1	1	28				1	1	13	2	2	25
Q8IV33	>sp|Q8IV33|K0825_HUMAN Uncharacterized protein KIAA0825 OS=Homo sapiens GN=KIAA0825 PE=2 SV=2	2	1	1	1	1	1							1	1	1	1	1	1			
O95258	>sp|O95258|UCP5_HUMAN Brain mitochondrial carrier protein 1 OS=Homo sapiens GN=SLC25A14 PE=2 SV=1	1	1	3	1	1	3							1	1	3						
P30041	>sp|P30041|PRDX6_HUMAN Peroxiredoxin-6 OS=Homo sapiens GN=PRDX6 PE=1 SV=3	1	1	4				1	1	4	1	1	4									
P62834	>sp|P62834|RAP1A_HUMAN Ras-related protein Rap-1A OS=Homo sapiens GN=RAP1A PE=1 SV=1				1	1	7	1	1	7	1	1	7	1	1	7	1	1	7			
Q13867	>sp|Q13867|BLMH_HUMAN Bleomycin hydrolase OS=Homo sapiens GN=BLMH PE=1 SV=1										1	1	2	1	1	2				1	1	4
Q5D862	>sp|Q5D862|FILA2_HUMAN Filaggrin-2 OS=Homo sapiens GN=FLG2 PE=1 SV=1							1	1	2	1	1	2	1	1	2	1	1	2	1	1	2
Q6P2C0	>sp|Q6P2C0|WDR93_HUMAN WD repeat-containing protein 93 OS=Homo sapiens GN=WDR93 PE=2 SV=1	1	1	2	1	1	2	1	1	2	1	1	2			2	1	1	2	1	1	2
Q9BTE3	>sp|Q9BTE3|MCMBP_HUMAN Mini-chromosome maintenance complex-binding protein OS=Homo sapiens GN=MCMBP				1	1	2	1	1	2	1	1	2	1	1	2						

Supplementary Table S2.- Protein abundance changes in HDL proteome from AAA vs control patients.

Gene name	Accession number[1]	Protein name	*Xq* = Log_2 (AAA/control)																	
			Individual comparatives														Integration data			
			1	2	3	4	5	6	7	8	9	10	11	12	13	14	*Xq*	*Zq*	*p-value*	*FDR*
PRDX6	**P30041**	**PRDX6_HUMAN Peroxiredoxin-6**	1.12		0.77	1.78				1.59		1.82	0.58				**1.29**	**3.79**	**0.0002**	**0.0116**
RBP4	**P02753**	**RET4_HUMAN Retinol-binding protein 4**	1.71	2.32	1.02	1.45	1.43	1.89	2.26	0.35	0.71	0.75	0.69	0.82	-0.77	-0.29	**1.03**	**3.59**	**0.0003**	**0.0176**
HLA-A	**P30447**	**1A23_HUMAN HLA class I histocompatibility antigen, A-23 alpha chain**	-1.98		-0.53		1.26	1.83		-1.65		1.34		3.61	2.74		**1.13**	**3.51**	**0.0004**	**0.0211**
PON1	**P27169**	**PON1_HUMAN Serum paraoxonase/arylesterase 1**	0.16	1.24	1.88	0.95	1.02	1.22	1.55	0.57	0.47	0.03	1.74	1.58	-0.05	1.01	**0.95**	**3.35**	**0.0008**	**0.0309**
PFN1	P07737	PROF1_HUMAN Profilin-1	1.16		0.11	1.30	2.04	1.24	-0.39	1.10		1.57	0.48	1.12	0.14	1.10	0.90	3.04	**0.0024**	0.0730
CST3	P01034	CYTC_HUMAN Cystatin-C	0.42	1.56	1.09	1.19	1.38	2.82	2.66	0.69	0.66	1.01	0.54	1.11	1.69	1.37	0.87	2.98	**0.0029**	0.0847
SAA	E9PQD6	E9PQD6_HUMAN Serum amyloid A protein	-0.31	1.82	-0.65					1.52	0.00	-0.28					0.94	2.78	**0.0055**	0.1454
ACTA2	P62736	ACTA_HUMAN Actin, aortic smooth muscle	0.31	0.16	-0.06	1.10	1.10	2.23	-1.03	1.25	1.31	1.87	0.82	1.47	2.28	0.93	0.77	2.69	**0.0072**	0.1724
PF4	P02776	PLF4_HUMAN Platelet factor 4			0.06	0.65	0.51	1.84				1.90	0.90	-0.23	0.40		0.79	2.55	**0.0108**	0.2370
CNDP1	Q96KN2	CNDP1_HUMAN Beta-Ala-His dipeptidase	1.41	0.99	-1.65	1.41	1.41	1.27	1.62	0.16	0.54	-0.36	0.62	-0.02	-0.53	0.48	0.72	2.52	**0.0117**	0.2474
LPA	Q1HP67	Q1HP67_HUMAN Lipoprotein, Lp(A)	2.36	3.39		-2.12		0.10		-0.60	0.16				-0.17		0.79	2.44	**0.0148**	0.2893
SERPINF1	P36955	PEDF_HUMAN Pigment epithelium-derived factor	0.83	1.32	0.66	1.59	1.92	1.78	1.34	-0.30	0.18	0.04	0.01	0.45	-0.60	-0.16	0.64	2.28	**0.0226**	0.3507
GC	P02774	VTDB_HUMAN Vitamin D-binding protein	0.58	1.08	0.61	1.15	2.17	1.52	1.96	-0.16	-0.21	0.11	-0.15	0.34	0.00	-0.10	0.64	2.27	**0.0235**	0.3539
LOC613037	A8MRT5	K220L_HUMAN Putative NPIP-like protein LOC100132247		1.50	0.73		0.66	0.15			1.47	0.44		0.92	-1.78		0.69	2.18	**0.0294**	0.4082
PPBP	P02775	CXCL7_HUMAN Platelet basic protein	0.60	0.43	0.12	0.44	0.84	1.44	-0.07	1.55	0.04	1.20	0.38	-0.19	1.14	0.37	0.60	2.13	**0.0331**	0.4364
B2M	P61769	B2MG_HUMAN Beta-2-microglobulin	0.41	1.55	0.63	0.29	0.65	0.36	0.16	0.24	0.63	1.04	0.06	0.46	1.77	0.01	0.61	2.12	**0.0339**	0.4253
LCAT	P04180	LCAT_HUMAN Phphatidylcholine-sterol acyltransferase	0.51	1.05	0.56	0.73	1.15	1.58	2.23	0.13	0.46	0.02	0.46	0.96	-0.45	0.56	0.58	2.08	**0.0372**	0.4458
AMBP	P02760	AMBP_HUMAN Protein AMBP	0.89	1.37	0.87	0.47	1.34	1.31	1.23	-0.14	0.12	0.58	-0.25	0.31	0.41	-0.36	0.57	2.04	**0.0411**	0.4608
RAP1A	P62834	RAP1A_HUMAN Ras-related protein Rap-1A		-0.09	0.00	2.10	1.24	0.52			0.86	3.33	1.52	1.00	-2.12		0.65	1.98	**0.0479**	0.5151
LBP	P18428	LBP_HUMAN Lipopolysaccharide-binding protein	0.36	0.14	0.68	1.25	1.46	0.57	0.36	0.17	0.72	-0.11	0.42	1.46	0.28	0.32	0.55	1.94	0.0530	0.5582
N/A	B3KNA1	B3KNA1_HUMAN cDNA FLJ14021 fis, clone HEMBA1002513, highly similar to Histone deacety	0.16	-0.25					0.24	0.61	-0.28					-0.55	0.62	1.87	0.0611	0.5853
PGBD3	Q8N328	PGBD3_HUMAN PiggyBac transposable element-derived protein 3		0.66	1.22			1.22			0.27	0.02			0.10		0.59	1.78	0.0755	0.6522
ACTB	P60709	ACTB_HUMAN Actin, cytoplasmic 1	-0.08	0.24	-0.17	0.88	0.53	0.43	-0.67	0.88	0.79	1.22	0.69	0.62	0.85	0.75	0.47	1.68	0.0928	0.7193
SAA	P0DJI8	SAA1_HUMAN Serum amyloid A-1 protein	1.86	1.60	-1.97	0.23	-0.22	-0.71	-0.44	0.45	0.33	-0.58	0.17	0.40	2.24	-0.11	0.44	1.59	0.1124	0.8227
N/A	Q15994	Q15994_HUMAN Pyruvate dehydrogenase alpha subunit (Fragment)	-0.15	0.44			1.29	1.31	1.53	-0.06	0.09			0.25	0.16	0.20	0.49	1.59	0.1128	0.8145
PLA2G7	Q13093	PAFA_HUMAN Platelet-activating factor acetylhydrolase	1.63	0.95	0.97	-0.47	-0.86	-0.08	-0.62	0.23	0.37	0.74	0.47	0.48	1.24	1.07	0.43	1.54	0.1244	0.8192
PF4V1	P10720	PF4V_HUMAN Platelet factor 4 variant	-0.25	0.35	-0.09	0.10	0.38	1.62		0.47	0.33	0.60	0.18	0.46	-0.07		0.42	1.47	0.1411	0.8171
S100A9	P06702	S10A9_HUMAN Protein S100-A9		1.27		-0.04		-0.99	0.13		1.98		0.07		1.06	0.36	0.43	1.43	0.1523	0.8541
AHSG	P02765	FETUA_HUMAN Alpha-2-HS-glycoprotein	0.51	0.95	1.15	0.85	1.20	-0.25	1.37	-0.47	-0.06	-0.17	-0.07	-0.05	0.07	-0.04	0.35	1.33	0.1838	0.9139
HLA-A	P30443	1A01_HUMAN HLA class I histocompatibility antigen, A-1 alpha chain	-2.77	-0.33	0.06	1.21	1.04	1.20	0.02	-0.88	0.08	1.64	0.39	2.37	1.82	-0.19	0.33	1.21	0.2263	0.9391
UBA52	P62987	RL40_HUMAN Ubiquitin-60S ribosomal protein L40		0.37	0.20	0.16	-0.21	1.35	0.92		0.32	0.26	0.04	0.90	-0.47	0.03	0.33	1.18	0.2381	0.9578
ARHGEF17	Q96PE2	ARHGH_HUMAN Rho guanine nucleotide exchange factor 17	0.98		1.68	-0.50				0.05		0.27	-0.02				0.37	1.17	0.2409	0.9547
LYZ	P61626	LYSC_HUMAN Lysozyme C		0.80	0.24	-0.02	0.86		0.39		0.73	0.20	0.11	-0.04		0.00	0.33	1.16	0.2465	0.9623

Gene name	Accession number[1]	Protein name	1	2	3	4	5	6	7	8	9	10	11	12	13	14	Xq	Zq	p-value	FDR
			X_q = Log_2 (AAA/control)																	
			Individual comparatives														Integration data			
ALB	P02768	ALBU_HUMAN Serum albumin	-0.08	1.25	0.51	1.74	2.05	4.76	3.86	0.02	0.18	-0.22	-0.53	0.66	-0.33	-5.91	0.28	1.08	0.2817	1.0031
SAA	D3DQX7	D3DQX7_HUMAN Serum amyloid A protein	0.36		-0.86	-0.24	0.08	-0.23	-0.66	1.15		-0.23	0.63	0.70	-0.31	0.47	0.29	1.05	0.2946	0.9826
APOA4	P06727	APOA4_HUMAN Apolipoprotein A-IV	0.50	1.11	0.73	0.59	1.00	0.74	0.21	-0.13	-0.19	0.18	-0.25	-0.04	-0.13	-0.60	0.26	1.04	0.2976	0.9804
HBA1	P69905	HBA_HUMAN Hemoglobin subunit alpha	-0.41	-0.45	0.54	-0.02	0.29	0.37	0.25	0.62	0.02	0.05	0.09	0.20	2.55	-0.06	0.26	1.00	0.3196	0.9967
CETP	P11597	CETP_HUMAN Cholesteryl ester transfer protein		0.91	1.40	0.79	0.47	0.54	1.08		0.28	-0.96	-0.31	0.61	-3.82	-1.74	0.26	0.96	0.3350	1.0031
SAA2	P0DJI9	SAA2_HUMAN Serum amyloid A-2 protein	1.44	0.55	-1.10	0.64		-1.10		0.34	-0.02	-0.59	-0.14		0.86		0.26	0.95	0.3408	1.0146
APOD	P05090	APOD_HUMAN Apolipoprotein D	-0.05	0.31	0.35	-0.09	-0.13	-0.08	-0.18	0.30	0.67	0.46	0.03	0.53	0.80	0.78	0.23	0.95	0.3438	1.0179
HPR	P00739	HPTR_HUMAN Haptoglobin-related protein	-1.34	0.41	-0.41	0.51	0.83	1.33	1.51	-1.12	0.17	-0.15	0.71	0.61	-0.66	0.62	0.22	0.89	0.3744	1.0068
PRKACG	P22612	KAPCG_HUMAN cAMP-dependent protein kinase catalytic subunit gamma	-0.13	0.68					0.73	-1.84	-0.50					0.02	0.28	0.88	0.3805	0.9976
C9	P02748	CO9_HUMAN Complement component C9	0.66	-0.09	-0.66	0.69	0.67	-0.16	-0.32	0.34	0.24	0.00	0.42	0.33	0.45	0.49	0.20	0.84	0.4024	1.0195
SREBF2	Q12772	SRBP2_HUMAN Sterol regulatory element-binding protein 2	-0.12			0.19	1.15			0.03			-0.11	0.31			0.24	0.79	0.4290	1.0004
BPIFB1	Q8TDL5	BPIB1_HUMAN BPI fold-containing family B member 1	-0.34	0.09	0.66	0.18	0.69	0.13	0.96	0.07	0.94	-0.81	0.00	0.82	-0.41	0.62	0.20	0.79	0.4314	0.9972
PIBF1	Q8WXW3	PIBF1_HUMAN Progesterone-induced-blocking factor 1	0.12	0.22		-0.13	-0.60	-0.36	-0.48	0.45	0.95		0.14	0.54	0.73	0.27	0.18	0.71	0.4775	0.9869
APOC2	P02655	APOC2_HUMAN Apolipoprotein C-II	-0.19	0.15	0.53	-0.11	0.06	-0.58	0.02	-0.51	0.48	-0.31	0.73	1.09	0.02	0.72	0.15	0.68	0.4994	1.0007
SFTPB	P07988	PSPB_HUMAN Pulmonary surfactant-associated protein B	-0.41	-0.92	0.54	-1.09	0.03	0.62	-0.66	0.56	2.31	0.17	-0.50	1.44	0.45	-0.54	0.16	0.67	0.5000	0.9981
MENT	Q9BUN1	MENT_HUMAN Protein MENT	-0.60	-0.18		0.02	0.07	-0.41	-0.17	0.15	0.43		0.46	1.30	0.37	0.77	0.17	0.67	0.5044	0.9994
FAM117A	Q9C073	F117A_HUMAN Protein FAM117A			2.25		-1.13	-0.89				0.71		0.22	0.73		0.18	0.63	0.5270	0.9813
AZGP1	P25311	ZA2G_HUMAN Zinc-alpha-2-glycoprotein		0.93		0.67	0.84	0.75	0.22		0.03		-0.44	-1.59	0.39	-0.72	0.16	0.62	0.5376	0.9670
ITIH4	Q14624	ITIH4_HUMAN Inter-alpha-trypsin inhibitor heavy chain H4	-1.14	-1.71	-0.27	2.08	1.07	2.52		-0.38	0.63	-1.53	-0.19	-0.65	-2.55		0.12	0.52	0.6012	1.0122
ORM1	P02763	A1AG1_HUMAN Alpha-1-acid glycoprotein 1	-0.71	0.42	-0.52	-1.42	1.14	1.69	0.89	-0.35	-0.07	-0.22	-1.77	0.44	-0.67	-0.09	0.12	0.52	0.6015	1.0095
HBB	P68871	HBB_HUMAN Hemoglobin subunit beta	-0.02	0.08	2.12	0.12	0.18	-0.25	0.00	1.00	-0.03	0.01	0.53	-0.49	3.29	0.93	0.11	0.50	0.6155	1.0074
APOF	Q13790	APOF_HUMAN Apolipoprotein F	0.41	-0.04	0.07	0.18	-0.26	-0.52	-0.51	0.57	0.42	0.74	-0.22	0.19	0.29	0.70	0.11	0.50	0.6197	0.9896
ORM2	P19652	A1AG2_HUMAN Alpha-1-acid glycoprotein 2	-0.49	-0.15	0.03	0.35	0.73	1.63	1.42	-0.22	-0.31	-0.22	0.32	-0.07	-0.32	-0.35	0.09	0.42	0.6745	1.0098
FAM76B	Q5HYJ3	FA76B_HUMAN Protein FAM76B	0.46		0.00	-0.06	-0.01	-0.39		0.38		-0.04	0.44	0.81	-1.16		0.09	0.40	0.6868	0.9998
HELZ2	Q9BYK8	PR285_HUMAN Peroxisomal proliferator-activated receptor A-interacting complex 285 kDa p	0.32	-0.44		0.03	-0.66			0.51	0.22		0.68	-0.05			0.09	0.40	0.6906	1.0026
S100A8	P05109	S10A8_HUMAN Protein S100-A8			-0.34	0.12	-0.42		0.46			-0.58	-0.13	1.35		0.75	0.09	0.39	0.6951	1.0036
OLR1	P78380	OLR1_HUMAN Oxidized low-density lipoprotein receptor 1				0.38	0.03	0.09	0.50				0.95	-0.28	-0.85	-0.47	0.08	0.37	0.7143	0.9985
PON3	Q15166	PON3_HUMAN Serum paraoxonase/lactonase 3	0.04	0.33	0.18	-0.07	-0.54	-0.25	-0.15	0.23	0.42	0.61	-0.09	-0.01	0.27	-0.06	0.05	0.33	0.7432	1.0017
TTR	P02766	TTHY_HUMAN Transthyretin	0.12	0.55	0.03	0.62	0.47	0.86	0.98	-0.71	-0.37	-0.30	-0.22	-0.59	-0.38	-0.34	0.04	0.29	0.7703	0.9974
C4A	P0C0L4	CO4A_HUMAN Complement C4-A	-0.85	-0.02	0.41	0.32	0.18	3.60	0.58	0.16	0.12	0.06	-0.13	-0.11	-0.19	-0.58	0.03	0.25	0.8046	1.0072
SAA	B2R5G8	B2R5G8_HUMAN Serum amyloid A protein	0.71	0.79	-0.21	-0.20	-0.82	-0.39	-0.67	0.62	-0.21	0.11	0.13	-0.46	1.06	0.01	0.03	0.24	0.8128	1.0056
APOC3	P02656	APOC3_HUMAN Apolipoprotein C-III	-0.19	0.05	0.08	-0.18	-0.52	-0.14	-0.23	-0.12	0.43	0.01	0.07	0.58	0.59	0.09	0.03	0.23	0.8142	1.0049
CASP14	P31944	CASPE_HUMAN Caspase-14				0.17	-0.68		1.08				0.23	0.10		-0.62	0.03	0.20	0.8376	1.0009

			Xq = Log_2 (AAA/control)																	
			Individual comparatives														Integration data			
**Gene name**	**Accession number[1]**	**Protein name**	**1**	**2**	**3**	**4**	**5**	**6**	**7**	**8**	**9**	**10**	**11**	**12**	**13**	**14**	***Xq***	***Zq***	***p-value***	***FDR***
FGA	P02671	FIBA_HUMAN Fibrinogen alpha chain	-2.34	-1.76	-0.35	1.53	0.59	2.04	1.56	1.08	0.24	-0.73	0.00	-0.08	-0.36	-0.42	0.01	0.19	0.8516	1.0040
SAA4	P35542	SAA4_HUMAN Serum amyloid A-4 protein	0.42	0.48	-0.21	-0.32	-0.68	-0.54	-0.06	0.42	-0.85	0.24	0.09	-0.07	1.21	0.02	0.00	0.15	0.8771	1.0137
ANO9	A1A5B4	ANO9_HUMAN Anoctamin-9		0.47	-0.47				1.39		-0.19	-0.10				-0.31	0.00	0.12	0.9068	0.9977
SLC4A1	P02730	B3AT_HUMAN Band 3 anion transport protein	-0.44		0.00	0.03	-0.79	-0.94	1.16	1.36		0.76	0.52	-0.45	-1.19	-0.17	0.00	0.11	0.9106	0.9976
APOL1	O14791	APOL1_HUMAN Apolipoprotein L1	0.22	-0.05	0.39	-0.17	-0.39	-0.67	-0.20	-0.01	-0.56	0.57	-0.07	0.30	0.08	0.29	-0.02	0.08	0.9349	1.0076
HP	P00738	HPT_HUMAN Haptoglobin	-0.47	-1.65	0.31	1.04	2.35	0.86	-0.15	-0.84	-1.18	0.67	0.89	0.47	-1.58	1.26	-0.03	0.05	0.9629	1.0009
SELL	P14151	LYAM1_HUMAN L-selectin		0.11		0.58	0.04	-0.53			0.32		-0.32	-1.02	0.49		-0.04	0.01	0.9931	0.9969
CDH2	P19022	CADH2_HUMAN Cadherin-2	-0.19		1.11	0.04				-0.31		0.14	-0.57				-0.06	-0.04	0.9670	1.0012
APOE	P02649	APOE_HUMAN Apolipoprotein E	0.38	-0.44	1.28	-0.27	-0.80	-0.74	-0.90	-0.02	0.04	0.16	0.06	0.07	0.44	-0.14	-0.07	-0.10	0.9223	1.0043
C4B	Q6U2E9	Q6U2E9_HUMAN C4B1	-0.17	0.20	-0.48	0.07	-0.25	-0.08	-0.27	-0.23	0.29	0.49	0.12	0.01	0.21	-0.92	-0.08	-0.13	0.8973	1.0125
PCYOX1	Q9UHG3	PCYOX_HUMAN Prenylcysteine oxidase 1	0.18	0.23	0.45	-0.24	-0.52	-0.32	-0.13	-0.18	0.27	-0.09	0.13	0.06	-1.24	0.20	-0.08	-0.14	0.8876	1.0081
CYLD	Q9NQC7	CYLD_HUMAN Ubiquitin carboxyl-terminal hydrolase CYLD	-0.20	-0.34	-0.25					0.15	0.12	0.04					-0.09	-0.15	0.8836	1.0101
SERPINA1	P01009	A1AT_HUMAN Alpha-1-antitrypsin	-0.09	0.74	0.37	0.06	0.97	0.22		-1.51	-0.44	-0.22	-1.21	-0.38	-1.25		-0.09	-0.16	0.8717	1.0141
APOC1	P02654	APOC1_HUMAN Apolipoprotein C-I	0.15	-0.84	0.32	-0.50	-0.65	-0.38	-0.47	-0.38	-0.11	-0.15	-0.11	0.62	1.13	-0.41	-0.12	-0.26	0.7947	0.9995
MUC1	Q7Z542	Q7Z542_HUMAN Mucin short variant SV4		0.09			-0.62	-0.64			0.20			-0.33	0.47		-0.14	-0.27	0.7900	1.0008
KIAA0825	Q8IV33	K0825_HUMAN Uncharacterized protein KIAA0825	-0.17	0.01			-0.42	-1.50		-0.52	0.54			0.54	0.54		-0.13	-0.27	0.7845	0.9987
PRO2289	Q9P1D8	YP008_HUMAN Putative uncharacterized protein PRO2289		1.25	-0.31		0.63				-0.91	-0.65		-0.89			-0.15	-0.30	0.7667	1.0026
APOM	O95445	APOM_HUMAN Apolipoprotein M	0.03	-0.34	0.47	-0.34	-0.67	-0.51	-0.78	0.13	0.05	0.12	0.09	-0.34	0.31	0.10	-0.13	-0.30	0.7617	1.0035
LPA	P08519	APOA_HUMAN Apolipoprotein(a)	0.95	0.16	-1.37	0.64	1.09	0.25	-2.14	-1.87	-2.20	0.79	0.87	1.82	-0.01	0.85	-0.13	-0.32	0.7516	1.0028
AHNAK	Q09666	AHNK_HUMAN Neuroblast differentiation-associated protein AHNAK	-0.06	-0.52			-0.57			0.43	-0.35			0.19			-0.15	-0.32	0.7486	1.0013
ANTXR1	Q9H6X2	ANTR1_HUMAN Anthrax toxin receptor 1	-0.41		-0.38	0.03				0.33		-0.40	0.04				-0.16	-0.33	0.7409	1.0037
APOC4	P55056	APOC4_HUMAN Apolipoprotein C-IV	0.01	-0.61	-0.36	-0.42	-0.53	-0.09	-0.68	-0.13	-0.17	-0.15	0.01	0.96	0.65	-0.48	-0.15	-0.36	0.7195	1.0005
PIP	P12273	PIP_HUMAN Prolactin-inducible protein			-0.05	-0.61	-0.94					-0.31	-0.08	-0.40			-0.21	-0.45	0.6505	1.0024
CASC1	Q6TDU7	CASC1_HUMAN Cancer susceptibility candidate protein 1	0.18	0.50		0.08	-0.35	0.18		-0.80	-0.70		-1.11	0.27	0.01		-0.19	-0.46	0.6467	1.0084
FLG2	Q5D862	FILA2_HUMAN Filaggrin-2			0.17	-0.27	-0.69	-0.83	-0.37			-0.38	0.35	-0.07	0.20	0.00	-0.20	-0.50	0.6158	1.0046
APOA1	P02647	APOA1_HUMAN Apolipoprotein A-I	0.17	0.97	-0.36	-0.11	1.00	-0.80	-0.76	0.20	-0.21	-0.29	0.28	-0.03	0.16	0.03	-0.19	-0.52	0.6018	1.0069
APOB	C0JYY2	C0JYY2_HUMAN Apolipoprotein B (Including Ag(X) antigen)	-0.07	-0.41	1.05	-0.60	0.14	-0.04	-1.08	-0.29	0.18	0.09	-0.86	-0.79	-0.07	0.03	-0.21	-0.57	0.5702	0.9724
DCD	P81605	DCD_HUMAN Dermcidin	-0.13		-0.03	0.59			0.10	-0.05		0.25	-0.07			-0.11	-0.23	-0.57	0.5663	0.9689
SERPINF2	P08697	A2AP_HUMAN Alpha-2-antiplasmin	-0.04		-0.08		-0.55			-0.04		-0.13		-0.65			-0.26	-0.62	0.5338	0.9701
VTN	P04004	VTNC_HUMAN Vitronectin	0.25	-0.06	-0.35	0.21	-0.20	-0.19	-0.04	-0.51	-0.41	-0.52	-0.29	-0.19	-0.46	-0.28	-0.23	-0.63	0.5264	0.9837
FADS1	O60427	FADS1_HUMAN Fatty acid desaturase 1	0.21	-0.62		-0.32	-0.55	-1.08	-1.02	-0.25	-0.01		-0.28	-0.11	0.26	0.45	-0.24	-0.64	0.5239	0.9826
SLC25A14	O95258	UCP5_HUMAN Brain mitochondrial carrier protein 1	-0.13	1.31		-0.11	0.64	0.05		-2.53	-0.25		-1.87	-0.96	-2.61		-0.28	-0.69	0.4874	0.9879
HABP4	Q5JVS0	HABP4_HUMAN Intracellular hyaluronan-binding protein 4 OS					-0.62	-0.99	-0.55					0.29	0.09	-0.05	-0.29	-0.70	0.4823	0.9851
NDEL1	Q9GZM8	NDEL1_HUMAN Nuclear distribution protein nudE-like 1	-0.40	-0.39	-0.36					-0.07	0.27	-0.49					-0.29	-0.71	0.4767	0.9891

			Xq = $\log_2$ (AAA/control)																	
			**Individual comparatives**														**Integration data**			
**Gene name**	**Accession number**[1]	**Protein name**	**1**	**2**	**3**	**4**	**5**	**6**	**7**	**8**	**9**	**10**	**11**	**12**	**13**	**14**	***Xq***	***Zq***	***p-value***	***FDR***
RANBP2	P49792	RBP2_HUMAN E3 SUMO-protein ligase RanBP2				-0.67	-0.58	-0.59					-0.02	-0.10	0.25		-0.31	-0.74	0.4610	0.9835
ANTXR2	P58335	ANTR2_HUMAN Anthrax toxin receptor 2	-0.66	-0.19		-0.46	-0.71	-0.46	-0.92	-0.04	0.43		-0.04	-0.51	0.26	-0.08	-0.27	-0.76	0.4487	0.9853
RELL2	Q8NC24	RELL2_HUMAN RELT-like protein 2	-0.05	-1.04	1.13			-0.90		-0.25	-1.04	0.17			0.02		-0.31	-0.81	0.4199	1.0014
P2RY12	B2RE67	B2RE67_HUMAN cDNA, FLJ94014, highly similar to Homo sapiens purinergic receptor P2Y				-0.47	-0.84	-0.18					-0.24	-0.33	0.14		-0.34	-0.82	0.4123	1.0012
PRSS1	P07477	TRY1_HUMAN Trypsin-1	-0.57	-0.59	-0.55					-0.09	0.09	0.01					-0.32	-0.82	0.4095	1.0131
SERPING1	P05155	IC1_HUMAN Plasma protease C1 inhibitor	0.35	-0.91	-1.30	-0.36	-1.15	1.25	-0.42	1.15	0.40	-0.51	-0.86	-0.51	-0.06	-1.06	-0.29	-0.84	0.3983	1.0140
SERPINA10	Q9UK55	ZPI_HUMAN Protein Z-dependent protease inhibitor				-0.09	-1.11	0.70					-0.08	-1.24	-0.81		-0.34	-0.85	0.3950	1.0154
CLU	P10909	CLUS_HUMAN Clusterin	-0.22	-0.39	0.00	-0.17	-0.27	-0.56	-0.30	-0.04	-0.08	-0.36	-0.44	-0.36	-0.33	-0.56	-0.30	-0.90	0.3666	1.0223
IGKC	P01834	IGKC_HUMAN Ig kappa chain C region	0.06	-0.82	0.15	-0.38	-0.40	-0.94	-0.66	-0.19	-0.57	0.59	-0.31	-0.45	-0.06	-0.35	-0.31	-0.92	0.3556	1.0186
FGG	P02679	FIBG_HUMAN Fibrinogen gamma chain	-0.31	-1.47	-0.12	-0.10		-1.21		0.04	-0.21	0.57	-0.37		-0.35		-0.33	-0.97	0.3330	1.0028
DSP	P15924	DESP_HUMAN Desmoplakin				-0.44	0.07	-0.15	-1.08				0.04	0.15	0.09	-1.05	-0.38	-1.03	0.3050	0.9801
2 SV	A8K479	A8K479_HUMAN cDNA FLJ77094, highly similar to Homo sapiens apolipoprotein B	-0.78	-2.14		-0.23				2.02	0.49		-0.24				-0.38	-1.05	0.2936	0.9856
APOA2	P02652	APOA2_HUMAN Apolipoprotein A-II	-0.36	-0.64	0.03	-0.55	-1.15	-1.10	-0.69	0.19	-0.20	-0.17	0.23	-0.24	-0.06	-0.13	-0.35	-1.07	0.2825	0.9994
TRANK1	O15050	TRNK1_HUMAN TPR and ankyrin repeat-containing protein 1	-0.09	-0.80	-0.31	-0.68				-0.44	-0.20	-0.43	-0.41				-0.42	-1.14	0.2551	0.9671
AGT	P01019	ANGT_HUMAN Angiotensinogen	-1.02	0.39	-0.26	-0.45	1.24	0.87	1.37	-0.57	0.22	-0.74	-1.46	-0.95	-0.96	0.15	-0.38	-1.17	0.2433	0.9570
PLTP	P55058	PLTP_HUMAN Phpholipid transfer protein	-0.97	-0.40	0.19	-0.42	-0.42	-1.03	-0.40	0.16	0.40	-0.46	-0.28	-0.40	-0.31	-0.95	-0.39	-1.22	0.2214	0.9411
APOB	P04114	APOB_HUMAN Apolipoprotein B-100	-0.14	-0.48	1.16	-0.63	-0.27	-0.14	-3.34	-0.14	0.01	0.00	-0.80	-1.12	-0.32	1.28	-0.41	-1.30	0.1939	0.9126
IGHA1	P01876	IGHA1_HUMAN Ig alpha-1 chain C region	0.08	-1.10	0.53	-0.03	-0.28	-1.44	-0.96	-0.68	0.06	0.16	-0.40	-0.50	-0.66	-0.48	-0.43	-1.33	0.1845	0.9087
CAMP	P49913	CAMP_HUMAN Cathelicidin antimicrobial peptide	-0.27	-0.52	-0.49	-0.96	-1.64	-2.28		-0.10	-0.50	1.21	0.36	0.00	-0.67		-0.46	-1.34	0.1804	0.9144
C6orf211	F2Z3I8	F2Z3I8_HUMAN UPF0364 protein C6orf211	-0.18	-1.02		-0.83	0.03		-1.41	0.00	0.11		-0.78	-1.22		0.15	-0.49	-1.39	0.1652	0.8881
FGB	P02675	FIBB_HUMAN Fibrinogen beta chain	-0.55	-1.07	0.10	-0.81	-0.86	-1.32	-1.48	0.19	-0.14	0.74	-0.55	-0.28	-0.62	-0.39	-0.49	-1.50	0.1346	0.8153
IGHG2	P01859	IGHG2_HUMAN Ig gamma-2 chain C region	-0.18		-0.59	-0.56	-1.23	-1.67	-1.31	-0.50		-0.13	0.39	-1.02	0.36	-0.04	-0.52	-1.53	0.1263	0.8018
DSG1	Q02413	DSG1_HUMAN Desmoglein-1		1.42	-0.01	-0.32			-1.11		0.22	-0.29	0.05			-0.64	-0.54	-1.53	0.1259	0.8193
IGHG1	P01857	IGHG1_HUMAN Ig gamma-1 chain C region	-0.49	-1.46	0.17	-0.55	-0.60	-1.55	-1.71	-0.39	-0.26	1.44	-0.51	-0.16	-0.38	-0.35	-0.49	-1.56	0.1196	0.8078
APOH	P02749	APOH_HUMAN Beta-2-glycoprotein 1	-0.44	-0.72	0.06	-0.53	-0.78	-0.84	-0.38	0.09	0.00	1.29	-0.43	-0.71	-0.69	-1.26	-0.56	-1.75	0.0805	0.6632
IGLL5	B9A064	IGLL5_HUMAN Immunoglobulin lambda-like polypeptide 5	-0.12	-1.42		-0.24	-0.61	-1.10	-1.49	-0.61	-0.34		0.25	-0.98	-1.04	-0.45	-0.62	-1.90	0.0572	0.5685
MMRN2	Q9H8L6	MMRN2_HUMAN Multimerin-2 OS					-1.15	-1.05	-0.81					-0.54	-0.15	-0.34	-0.72	-1.91	0.0562	0.5698
C3	P01024	CO3_HUMAN Complement C3	-0.80	-0.38	-0.03	-0.61	-0.35	-0.71	-0.88	-0.63	-0.54	-0.68	-0.54	-0.77	-0.72	-7.16	-0.62	-2.04	**0.0412**	0.4523
C2orf15	Q8WU43	CB015_HUMAN Uncharacterized protein C2orf15	-0.01					-1.90	-2.74	-0.02					-0.17	-0.71	-0.85	-2.23	**0.0259**	0.3696
IGHM	P01871	IGHM_HUMAN Ig mu chain C region	-0.18	-0.62	-0.49	-0.13	-2.16	0.20	-1.66	-0.38	-1.18	-0.04	-0.23	-2.52	-0.06	-1.02	-0.71	-2.23	**0.0256**	0.3752
TF	P02787	TRFE_HUMAN Serotransferrin	-0.32	-1.04	-0.07		-1.04	-2.17	-1.84	-0.45	-1.01	-0.36		-0.99	-0.64	-0.50	-0.84	-2.66	**0.0079**	0.1800
C4BPA	**P04003**	**C4BPA_HUMAN C4b-binding protein alpha chain**	-1.50	-2.03		-1.54	-2.00		-1.21	-0.95	-1.22		-0.94	-1.36		-0.49	**-1.36**	**-4.26**	**0.0000**	**0.0018**
A2M	**P01023**	**A2MG_HUMAN Alpha-2-macroglobulin**	-0.89		0.03		-3.22	-1.86		-1.11		-0.26		-2.77	-1.33		**-1.49**	**-4.47**	**0.0000**	**0.0008**

-2.5 **-1.5** **0** **1.5** **3**

decrease in AAA   incease in AAA

Accession number from the Uniprot database (http://www.uniprot.org/).

Supplementary Table S3.- Statistical parameters estimated for all the AAA/IC and C/IC comparatives from all the iTRAQ experiments

iTRAQ Experiment	Comparative	Spectrum variance (σ_S) (95% C.I.*)	Peptide variance (σ_P) (95% C.I.)	Protein variance (σ) (95% C.I.)
iTRAQ 1	116/117	0.0325 (0.029-0.036)	0.0443 (0.034-0.052)	0.1896 (0.144-0.28)
	115/117	0.0424 (0.037-0.046)	0.059 (0.047-0.078)	0.0861 (0.045-0.134)
	114/117	0.0493 (0.043-0.055)	0.0293 (0.02-0.039)	0.16 (0.079-0.199)
iTRAQ 2	116/117	0.0307 (0.029-0.035)	0.0964 (0.078-0.117)	0.1969 (0.313-0.28)
	115/117	0.045 (0.041-0.049)	0.0556 (0.043-0.069)	0.2956 (0.195-0.39)
	114/117	0.0552 (0.051-0.061)	0.0223 (0.015-0.029)	0.1193 (0.077-0.188)
iTRAQ 3	116/117	0.0392 (0.036-0.043)	0.0427 (0.033-0.056)	0.05800 (0.031-0.099)
	115/117	0.0507 (0.045-0.056)	0.0575 (0.038-0.071)	0.0855 (0.05-0.124)
	114/117	0.0777 (0.065-0.087)	0.0492 (0.015-0.063)	0.1089 (0.068-0.17)
iTRAQ 4	116/117	0.0442 (0.04-0.049)	0.03740 (0.028-0.049)	0.2132 (0.152-0.29)
	115/117	0.0507 (0.045-0.055)	0.0325 (0.025-0.044)	0.0779 (0.053-0.111)
	114/117	0.0726 (0.063-0.079)	0.0333 (0.022-0.045)	0.2295 (0.153-0.315)
iTRAQ 5	116/117	0.0768 (0.07-0.085)	0.0546 (0.039-0.07)	0.3697 (0.237-0.5)
	115/117	0.0694 (0.064-0.077)	0.0474 (0.03-0.058)	0.2343 (0.149-0.348)
	114/117	0.0816 (0.075-0.088)	0.0693 (0.054-0.088)	0.8417 (0.619-1.183)
iTRAQ 6	116/117	0.1108 (0.099-0.123)	0.1813 (0.147-0.228)	0.647 (0.424-1.007)
	115/117	0.0742 (0.068-0.081)	0.0571 (0.039-0.069)	0.3932 (0.283-0.626)
	114/117	0.0969 (0.088-0.104)	0.0365 (0.023-0.048)	0.3592 (0.249-0.524)
iTRAQ 7	116/117	0.0548 (0.051-0.06)	0.0322 (0.022-0.042)	0.4106 (0.299-0.54)
	115/117	0.0907 (0.085-0.1)	0.0638 (0.047-0.078)	0.2023 (0.126-0.313)
	114/117	0.0928 (0.084-0.102)	0.0624 (0.037-0.08)	0.4557 (0.327-0.6519

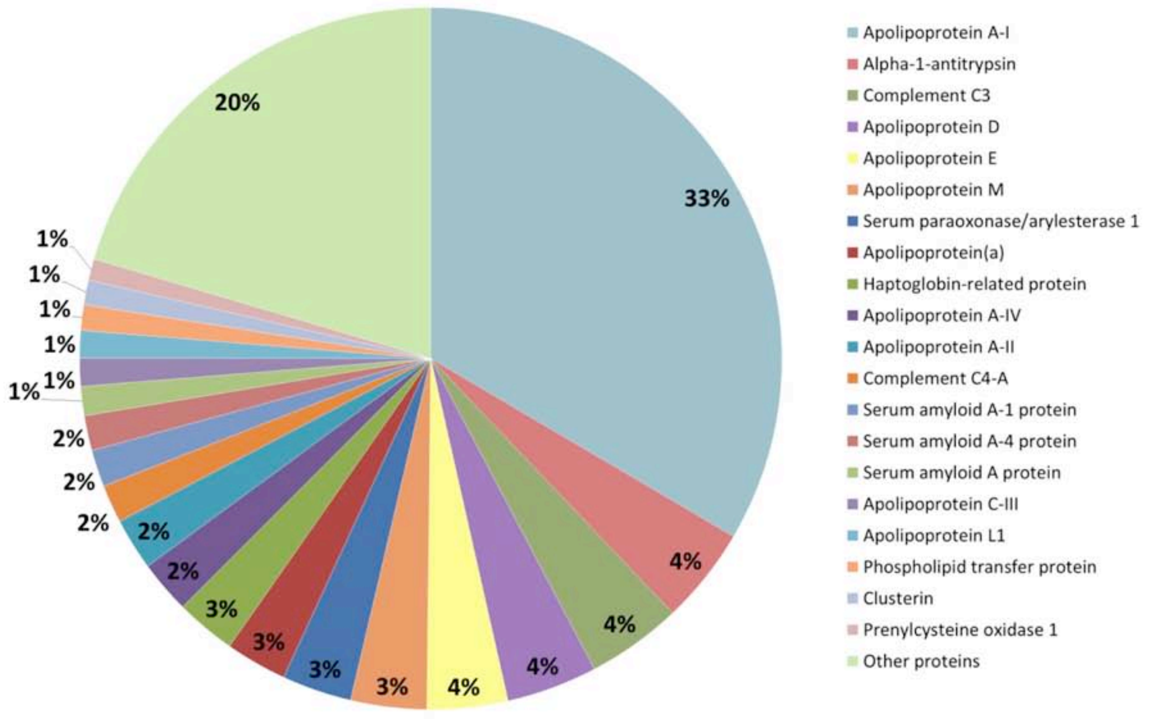

Supplementary Figure S1. Protein profile of HDL particle. Proteins were identified by LC-MS/MS in HDL samples isolated by ultracentrifugation from 7 donors and 14 AAA patients. Peptides were identified with 1% FDR and proteins identified in at least 3 experiments were counted.

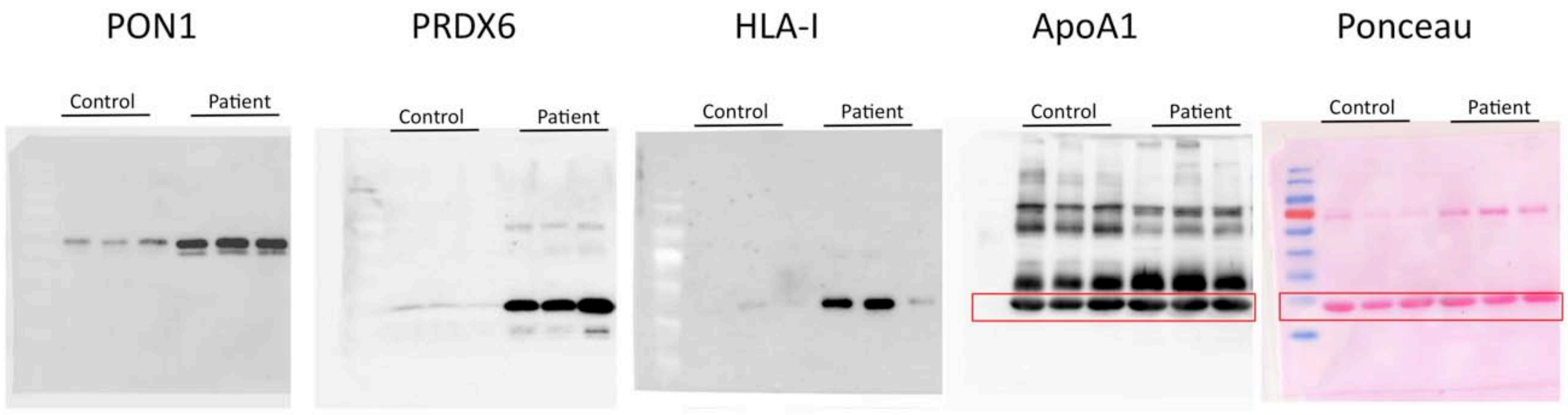

Supplementary Figure S2. Western-blot of validated proteins (full-image). Representative western-blot of PON1, PRDX6, HLA-I and APOA1, and Ponceau staining of HDLs from controls and patients. Red square indicates the band of ApoA1 used for densitometric analysis (based on their molecular weight) and used as loading control (since ApoA1 is the majority protein in HDL as observed in the ponceau staining).

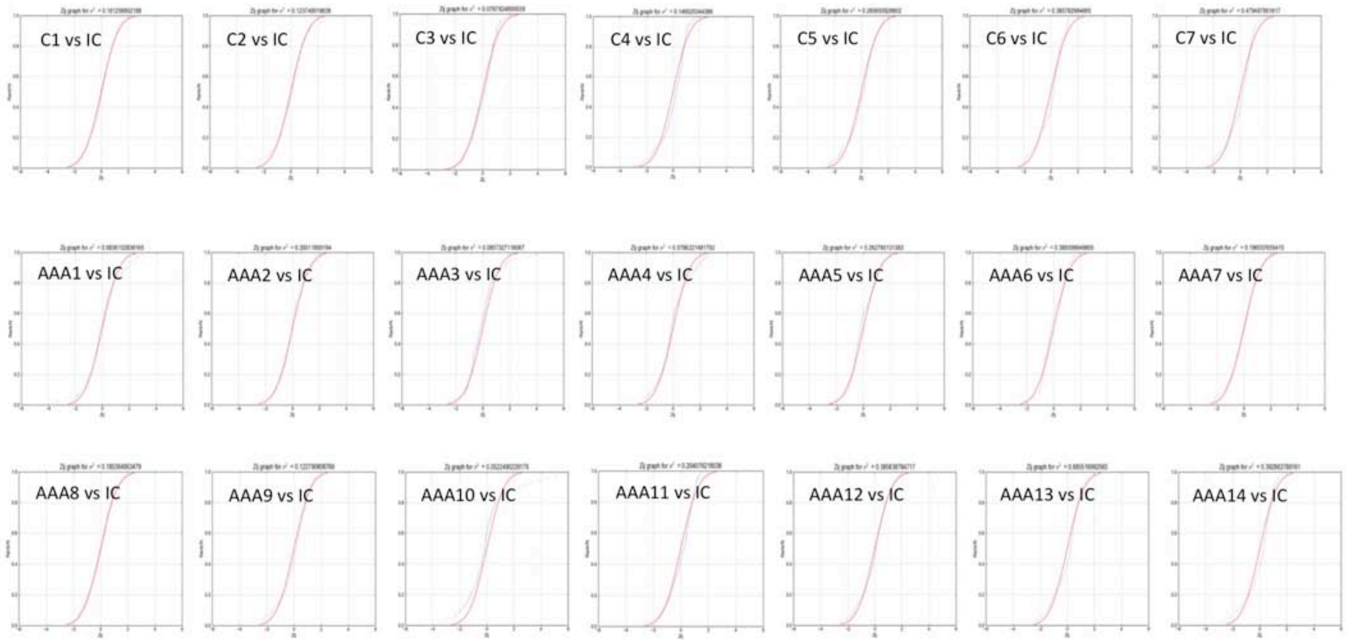

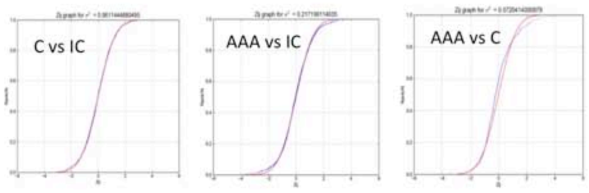

Supplementary Figure S3. Integrative analysis of the HDL protein abundance changes in AAA patients with respect to control subjects. Data from quantitative iTRAQ experiments comparing control subjects with the internal control (C1, C2, C3, C4, C5, C7, and C9 vs IC) were integrated to obtain average protein values for control (C) vs IC. Data comparing AAA patients with the internal control (AAA1, AAA2, AAA3, AAA4, AAA5, AAA6, AAA7, AAA8, AAA9, AAA10, AAA11, AAA12, AAA13, and AAA14 vs IC) were integrated to obtain average protein values for AAA vs IC. Finally, quantitative data for comparison of AAA vs C were obtained after integration of the comparisons of C vs IC and AAA vs IC. Plots represent the cumulative distribution of the standardized variable in logarithmic scale (log_2-ratios) at the different level of the integration data.

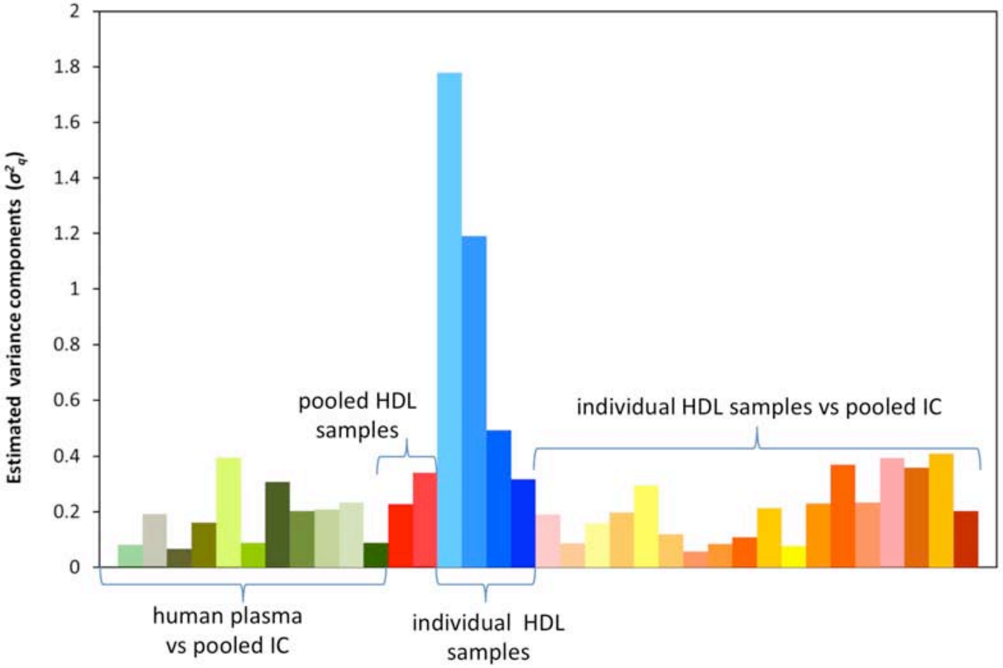

Supplementary Figure S4. Biological variability of individual and pooled samples in different experiments. Individual human plasma samples from AAA patients and control subjects were compared with a pooled internal control in different iTRAQ experiments (Burillo E, Lindholt JS, Molina-Sánchez P,et al. Thromb Haemost. 2015 May 26;113(6):1335-46) (green). Pooled HDL samples from CAD patients before and after angioplasty were compared in O16/O18 experiments (red). Individual HDL samples from two CAD patients before and after angioplasty were compared in different iTRAQ experiments (blue) (Jorge I, Burillo E, J. Proteomics, 2014). In the current iTRAQ study, individual HDL samples from AAA patients and control subjects were compared with the pooled internal control (yellow).
